# The Potency of Maillard Conjugates Containing Whey Protein as Natural Emulsifier

**DOI:** 10.1155/2024/3254132

**Published:** 2024-05-27

**Authors:** Iceu Agustinisari, Kamarza Mulia, Niken Harimurti, Mohammad Nasikin, Rienoviar, Heny Herawati, Lamhot Parulian Manalu

**Affiliations:** ^1^ Research Center for Agroindustry National Research and Innovation Agency KST Soekarno Cibinong, Jl. Raya Jakarta-Bogor KM 46, Cibinong 16911, Indonesia; ^2^ Department of Chemical Engineering Universitas Indonesia, Depok 16424, Indonesia

## Abstract

There is a continued need for the advancement of natural emulsifiers to replace synthetic emulsifiers, driven by human health concerns. This study is aimed at producing protein-polysaccharide conjugates through the Maillard reaction and at evaluating its ability as an emulsifier based on its emulsifying properties. The proteins used in this study were bovine milk whey protein and soy protein isolates, while the polysaccharides were maltodextrin and pectin. The protein-polysaccharide conjugation used a Maillard reaction under dry heating conditions. The protein and polysaccharide mass ratios were 1 : 2 and 1 : 3. The results showed that the types of proteins and polysaccharides and their mass affect the surface tension of the conjugate products. Whey protein-pectin conjugates with a mass ratio of 1 : 2 and a concentration of 1% had the lowest surface tension at 43.77 dyne/cm^2^. This conjugate sample also showed the highest emulsifying index at 27.20 m^2^/g. The conjugate powder containing pectin as a polysaccharide showed better emulsifying activity than that of those containing maltodextrin. However, the smallest droplet size of the emulsion (256.5 nm) resulted from the emulsification process using whey protein-maltodextrin conjugates as an emulsifier. The FTIR and gel electrophoresis (SDS-PAGE) analysis confirmed the conjugation formation. In general, protein-polysaccharide conjugates containing whey protein could potentially act as a natural emulsifier for food.

## 1. Introduction

Emulsions are widely used in the manufacturing processes of several industries, including the food, pharmaceutical, and nonfood sectors, such as paints, textiles, pesticides, and metal cutting fluids [1]. Generating a stable emulsion requires the input of energy [2]. Moreover, the use of supplementary surface-active compounds or emulsifiers is warranted. In general, emulsifiers used in food processing are synthetic, including fatty alcohol ethoxylates [3], sorbitan esters [4], and sucrose esters [5]. Synthetic emulsifiers may have potential toxicity to human health [6]. It is widely acknowledged that natural emulsifiers possess enhanced safety attributes and contribute to the improved acceptability of food products among consumers [7]. Due to these factors, there has been an increased focus on researching the advancement of natural emulsifier manufacturing. Proteins and polysaccharides are among the natural emulsifiers applied in the food industry [8].

The bovine milk whey protein is a waste product of the cheese and casein industry. The whey protein contains around 7-11% protein consisting of *β*-lactoglobulin, *α*-lactalbumin, bovine serum albumin, bovine lactoferrin, and immunoglobulins. Further purification of whey protein will produce whey protein isolate (WPI) and whey protein concentrate (WPC), which have more than 90% protein content. Other components of whey protein are lactose, carbohydrates, fat, and cholesterol, which are negligible in WPI [9]. In addition to essential nutrients, whey protein has functional properties such as water binding, gelling, emulsifying, foaming, and chelating capacities [9, 10]. These properties allow whey protein to act as an emulsifier.

Proteins and polysaccharides have properties that can emulsify and stabilise the emulsion. The amphiphilic nature of protein components can be used as an emulsifier to help reduce interfacial tension and stabilise the oil/water interface [11]. Protein exhibits sensitivity to pH variations, ionic strength, and temperature as a naturally occurring emulsifier. Dickinson [12] revealed that most polysaccharides have a stabilising ability by continuously forming tissue, making it highly viscous, enabling it to create a gel [13]. Combining both under certain conditions (concentration, protein-polysaccharide ratio, pH, ionic strength, and temperature) is a strategy to improve the stability of the emulsion [14].

The conjugation via the Maillard reaction is known to be induced by dry or wet heating. However, the dry heating method is more widely studied and has a higher reaction efficiency than the wet method [15]. The formation of conjugation occurs through a covalent linkage established between the carbonyl group present in the polysaccharide and the amine group located in the protein [16]. The Maillard reaction is a thermal process that begins with the production of a Schiff base, proceeds with the generation of ketamine from the Maillard reaction through the rearrangement of Amadori, and culminates in the development of melanoidin, which is distinguished by its brown pigment [17]. Melanoidin formation sometimes leads to undesirable conditions, such as bitter taste and burning aroma [18].

Several studies have demonstrated that the protein-polysaccharide conjugates enhance solubility and emulsifying capabilities to a greater extent than independent use of protein independently [19–21]. The formation of protein-polysaccharide conjugates is affected by the type and proportion of proteins and polysaccharides and processing conditions, including reaction time and temperature [17, 22]. This study used whey protein and soy protein, representing animal and plant protein, respectively. Furthermore, the use of whey protein and soy protein isolate will increase their added values because these proteins are waste products from the processing of cheese and soybean oil, respectively [23, 24]. According to Dammak et al. [6], there is a growing desire to encourage the exploration of novel natural emulsifiers sourced from industrial food by-products, which exhibit superior efficacy. Soy proteins derived from soybeans have been used extensively employed in food products due to their wide functional characteristics [25, 26]. Soybeans include several globular proteins with surface-active properties, which facilitate the creation of emulsions by lowering the interfacial tension between the oil and water phases.

Different types of protein-conjugated polysaccharides exhibit distinct impacts on the characteristics of the resulting conjugate product. Pectin represents a type of polysaccharide with hydrophobic groups and surface activity, while maltodextrin belongs to hydrophilic polysaccharides and is not surface active in an emulsion system [27]. Pectin is a branched polymer that has a linear backbone made up of *α*-D-galacturonic acid units with 1–4 linkages [28] obtained from various plant sources, most commonly citrus, apple, and beet [29], or extracted from food and beverage processing waste [30]. According to Peng et al. [31], maltodextrin exhibits a restricted capacity for adsorption and stabilisation of emulsion due to its lack of hydrophobic surface-active properties. Maltodextrin is derived through the acid and regulated enzymatic hydrolysis of starch, resulting in the formation of D-glucose polymers connected by *α*-(1,4) and *α*-(1,6) links [32, 33]. Maltodextrin is a stabiliser for oil-in-water food emulsions [34, 35]. A separate study has shown that the use of maltodextrin as a stabilising ingredient in oil-in-water emulsions can improve the physical stability of the emulsion [36].

Extensive research has been conducted on the maltodextrin conjugate whey protein isolate, revealing its promising capabilities in the emulsification of eugenol [20]. In addition, some studies have also been carried out on the conjugation of whey protein isolates with pectin [37]. Conjugate forming usually uses whey protein isolate (WPI) with a protein content of ±90%, while research that uses whey protein with 7-11% of protein is still rare. Another fact is that the whey protein isolate contains impurities such as lactose, which is also the case with the unrefined bovine milk whey protein. According to Ding et al. [22], lactose as a contaminant in WPI does not affect the formation of covalent bonds between WPI and maltodextrin. However, the effect of using whey protein in conjugation with polysaccharides is not yet known considering the difference in protein and impurity content. The successful formation of a conjugate based on whey protein will provide economic benefits and improve the added value of whey protein. Research has also been conducted on the conjugation of soy protein-maltodextrin through the Maillard reaction [38, 39]. Studies to examine the effects of different types of pectin on the production of conjugates were undertaken by Guo et al. [40] between pectin and whey protein during dry heating conditions. The Maillard reaction between soy protein and pectin derived from apple and citrus was investigated by Ma et al. [41].

Nevertheless, conducting research in this field is significant for acquiring optimal natural emulsifier products. In addition, the characteristics are contingent on the protein-polysaccharide ratio, process circumstances, and temperature. Proteins and polysaccharides are diverse, and the structural characteristics of these molecules greatly influence their emulsifying abilities. The objective of this study is twofold: first, to generate a protein-polysaccharide conjugate product through the process of whey protein/soy protein conjugation with maltodextrin/pectin through the dry heat Maillard reaction, and second, to evaluate and identify the optimal conjugate product based on its emulsifying properties.

## 2. Materials and Methods

### 2.1. Materials

The polysaccharides employed in this study were maltodextrin (technical grade) and pectin (technical grade). The proteins used were bovine milk whey protein (Sigma, W1500) and technical grade soy protein. The essential oil used for particle size measurement analysis was eugenol (Sigma-Aldrich, W246700). Gel electrophoresis used Mini-Protean SFX, 4-15%, 15 Well, 10 *μ*l, and the molecular weight protein marker was Precision Plus std dual protein from Bio-Rad Laboratories. The buffer solution for electrophoresis was prepared by reacting 62.5 mM Tris-HCl (Merck), 20% glycerol (Merck), 2% sodium dodecyl sulfate (Merck), and 0.05% bromophenol blue (Merck). The staining used Coomassie brilliant blue G250 (Sigma-Aldrich). Other chemicals used were absolute ethanol (Merck) and glacial anhydrous acetic acid for analysis (Merck).

### 2.2. Methods

#### 2.2.1. Preparation of Protein-Polysaccharide Conjugates through Maillard Reaction

**Figure 1:**
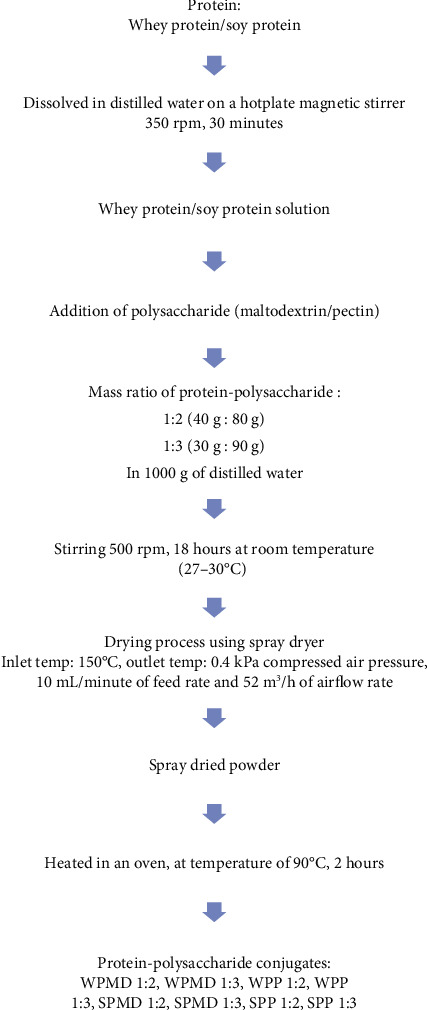
Flowchart of preparation of protein-polysaccharide conjugates through Maillard reaction.

The protein-polysaccharide conjugation method was adopted and modified from the Akhtar and Dickinson method [19] and Shah et al. [20]. The flow diagram for the preparation of protein-polysaccharide conjugates can be seen in Figure 1 and is described in detail as follows. Protein (whey protein or soy protein) was dissolved in distilled water and stirred with a magnetic bar on a hotplate magnetic stirrer at 350 rpm for 30 minutes until the protein was completely dissolved. Then, polysaccharide (maltodextrin or pectin) was added to the protein solution according to the formulation determined. The protein-polysaccharide mass ratios used in this study were 1 : 2 (40 : 80 g) and 1 : 3 (30 : 90 g), which were dissolved in 1000 g of distilled water. Shah et al. [20] stated that a mass ratio of protein and polysaccharides of 1 : 2 produces conjugates with the highest yield compared to mass ratios of 1 : 1 and 2 : 1. In addition to that, Capar and Yalcin [42] concluded that a protein-polysaccharide mass ratio of 1 : 2 is the optimum condition to produce the maximum yield. The stirring process was carried out at a speed of 500 rpm for 18 hours at room temperature (27-30°C) for the hydration process. The drying process was conducted using a spray dryer (Lab Plant Spray Dryer SD05) at an inlet temperature of 150°C and an outlet temperature of 80-90°C, 0.4 kPa compressed air pressure, 10 ml/minute of feed rate, and 52 m^3^/h of airflow rate. The use of temperatures above 150°C may damage the protein, considering that the denaturation temperature of whey protein or beta-lactoglobulin with low water content (aw 0.85-0.11) ranges from 96 to 180°C [43]. The spray-dried powder was heated in an oven cabinet at a temperature of 90°C for 2 hours for conjugation, and the Maillard reaction occurred. This research used the dry heating method, in which a mixture of protein powder and polysaccharides is heated at a specific temperature [9, 15]. The conjugate powders were collected in closed packaging and stored in the freezer (-12°C) until characterised. There are four combinations of protein-polysaccharide conjugates, namely, whey protein-maltodextrin (WPMD), whey protein-pectin (WPP), soy protein-maltodextrin (SPMD), and soy protein-pectin (SPP), and two mass ratios of protein-polysaccharide (1 : 2 and 1 : 3).

### 2.3. Characterisation

#### 2.3.1. Surface Tension Measurement

The measurement of the surface tension was done using a tensiometer with the Du Nouy ring method. This technique relies on dragging an object with a clearly defined geometry from the liquid surface while calculating the pull force. The Du Nouy method uses a ring as a pull object. This technique uses the contact between the platinum ring and the liquid surface. The ring is lowered below the interface by shifting the stage in which the liquid container is set up. After immersion, the stage gradually decreases as the ring raises the liquid's meniscus. This meniscus eventually separates from the ring. Before the tearing occurs, the volume of the meniscus (and the corresponding force) reaches its highest value and then starts to decrease (https://www.biolinscientific.com/). We investigated the effect of conjugate sample concentration (1% and 3%) on the surface tension of the solution. Determining of the concentration of the protein-polysaccharide conjugates tested was based on several types of literature. Emulsifiers play an important role in determining the stability of an emulsion, including surface tension and interfacial tension [44]. Jusoh and Othman [45] mentioned that the higher concentration of surfactant/emulsifier causes emulsion instability and concluded that 3% surfactant concentration can form a stable emulsion.

The dried powder of the protein-polysaccharide conjugates was dissolved in distilled water to make a solution of protein-polysaccharide conjugates. The mixture was homogenised with Ultraturrax (IKA T25) at 15,000 rpm for 1 minute. The measurement was made when no foam was on the solution's surface. The presence of foam will interfere with the accuracy of the surface tension measurement on the liquid surface. The tensiometer ring was placed below the surface of the liquid (±0.5 cm). Then, the ring was pulled upward through the surface of the liquid. The force needed for the ring to break the surface was expressed as the surface tension of the liquid (http://www.cscscientific.com/surface-tension/CSC-duNouy-Tensiometers). Measurements were taken 3 times for each sample.

#### 2.3.2. Emulsifying Activity Index (EAI)

The EAI procedure was based on the method described by Pearce and Kinsella (1978), as cited by Mu et al. [46]. Initially, the conjugate powder (0.2 g) was dissolved in 18 ml of a phosphate buffer (0.2 M, pH 7.4) that had been prepared in advance. In this test, we included a commercial emulsifier (Tween 80) as a control. Subsequently, oil-in-water emulsions were generated by introducing 5 ml of soy oil into each solution of the sample conjugate. The emulsions were homogenised at 15,000 rpm for 3 minutes using Ultraturrax T25 homogeniser (IKA, Germany). Then, 100 *μ*l of each emulsion was pipetted into 10 ml of 0.1% sodium dodecyl sulphate (SDS). The absorbance was measured at 500 nm. The EAI measurements for each sample were repeated three times. EAI was calculated using the following formula:

(1) $$\begin{array}{c} \text{EAI}=\frac{2T\times A\times \text{dilution factor}}{c\times Ф\times L\times 10000\;\left({\text{m}}^{2}/\text{g}\right)}, \end{array}$$

where *T* = 2.303, *A* is the absorbance, the dilution factor is 1000, *c* is the protein mass per volume unit (g/ml), *L* is the optical path wide (0.01 m), and *Ф* is the oil volumetric fraction (0.25).

#### 2.3.3. Particle Size, Polydispersity, and Zeta Potential Measurement

The particle size and zeta potential were measured to determine each conjugate sample's ability to emulsify eugenol with water to form an emulsion. The particle size and zeta potential were measured using a particle size analyser (Zetasizer, Malvern). The emulsifying ability of the conjugates was tested by adding protein-polysaccharide conjugates to the eugenol emulsion. The conjugate solution was prepared by dissolving 0.75 g of the conjugate sample in 25 ml of distilled water. Then, it was homogenised at 15,000 rpm for 1 minute using an Ultraturrax homogeniser (IKA, Germany). Eugenol (0.75 ml) was added to each sample solution homogenised at 15,000 rpm for 1 minute to obtain an emulsion. Three drops of emulsion (0.07 g) were diluted in 20 ml of distilled water in a glass beaker. Two millilitres of solution was poured into the cuvette for measurement. For each sample, measurements were taken 3 times.

#### 2.3.4. Thermal Property Analysis

The thermal properties of conjugate samples were analysed using the differential scanning calorimetry (DSC 8000 Perkin Elmer). The analysis was conducted on pure raw materials (whey protein, soy protein, maltodextrin, and pectin) and the resulting conjugate products. Each sample was weighed to approximately 7-10 mg and then prepared in an aluminium pan. The sample was heated from 40 to 300°C at a heating rate of 10.00°C/minute. Thermal transitions were evaluated in terms of peak transition temperature (Tp) and enthalpy (delta *H*). On the basis of the International Confederation of Thermal Analysis and Calorimetry Societies (ICTAC) standard, the peak value in the DSC thermogram is known as the melting point of the polymer sample (http://www.Perkinelmer.com).

#### 2.3.5. Fourier Transform Infrared (FTIR)

FTIR analysis was carried out to determine the molecular characteristics of each conjugate sample. The results of FTIR informed the possibility of changes in the formation of protein and polysaccharide conjugates. FTIR spectra of all samples were obtained using a Nicolet iS50 FTIR spectrometer (Thermo Scientific). The tool had a KBr beam splitter and a DTGS KBr detector. The spectra were recorded between 400 and 4000 cm^−1^ with a resolution of 2 cm^−1^. In addition to the conjugate samples, we included FTIR testing of whey protein, soy protein, maltodextrin, and pectin as controls.

#### 2.3.6. Sodium Dodecyl Sulphate-Polyacrylamide Gel Electrophoresis (SDS-PAGE) Analysis

SDS-PAGE is a method used to identify the formation of protein-polysaccharide conjugates. The research adopted and modified the procedures of Schmidt et al. [47] and Neirynck et al. [48] with modifications. The preparation of the conjugate samples was carried out by dissolving each sample in distilled water with a protein content of 4 mg/ml. Meanwhile, the whey protein and soy protein were dissolved with a protein content of 2 mg/ml as controls. The solution was vortexed for 1 minute until it formed a homogeneous solution. A 60 *μ*l solution was added to the vial with 20 *μ*l buffer solution (62.5 mM Tris-HCl, 20% glycerol, 2% SDS, and 0.05% bromophenol blue). The sample was heated in boiling water for 5 minutes until denaturation occurred in the conjugate solution. A total of 20 *μ*l for each sample solution was added to the 60 *μ*l buffer solution and applied to the gel, as well as the whey protein and the soy protein solution. A commercial molecular weight marker (Precision Plus std dual protein, Bio-Rad Laboratories) was also involved. Electrophoresis was carried out at 120 V by stacking (4% polyacrylamide) and separation (12% polyacrylamide) using Mini Cell Protean electrophoresis units (Bio-Rad Laboratories, USA). The process took 120 minutes. Then, staining was carried out with 0.25% (*v*/*v*) Coomassie brilliant blue in ethanol : acetic acid : water (45 : 10 : 45, *v*/*v*). The destaining process used an acetic acid solution : ethanol : water (5 : 10: 85, *v*/*v*).

#### 2.3.7. Scanning Electron Microscopy (SEM) Observation

The surface morphology of the conjugate dried powders was observed with a scanning electron microscope (ZEISS, EVO MA 10). The specimen holder/stainless stub was coated with carbon tape. The dry powder samples were placed and sprinkled as thin as possible on the surface of the carbon tape and then coated with gold by a sputter coating (Quorum Q150R ES) at sputter current conditions of 20 mA and sputter time of 60 seconds. The coated samples were mounted on the stage holder for SEM observation. The surface images were taken at different magnifications (100 to 5000x) using the SE (secondary electron) detector at working distance (WD) 9.0 mm and 16.00 kV accelerating voltages.

### 2.4. Statistical Analysis

All experiments except thermal property analysis, FTIR, and SDS-PAGE analysis were repeated three times. The results of the investigation were expressed as mean and standard deviation. Data were processed by analysis of variance (ANOVA), followed by the Tukey test to determine the significance between the average values, using MINITAB version 14.0. The level of significance was determined at *p* < 0.05. The graphs were created using Origin 2018 software (Originlab.com).

## 3. Results and Discussion

### 3.1. Surface Tension

#### 3.1.1. The Effect of Soy and Whey Protein in Conjugate Products Containing Maltodextrin on Surface Tension

The presence of an interfacial layer around the emulsion generally achieves the stability of a system. The properties of the interfacial layer are determined by the composition and structure of the adsorbed material, generally consisting of proteins and emulsifiers [49]. The interfacial tension between two liquids can be analogous to the surface tension or the tension at the interface of air and water. The hydrophobic nature of air makes the air-water and surfactant combination valuable for understanding the behaviour of surfactants. The reduction in surface tension serves as the initial criterion for determining the emulsification capacity of a candidate emulsifier [50]. A surface tension measurement was conducted to assess the efficacy of protein-polysaccharide conjugates as emulsifiers in decreasing surface tension.

**Figure 2:**
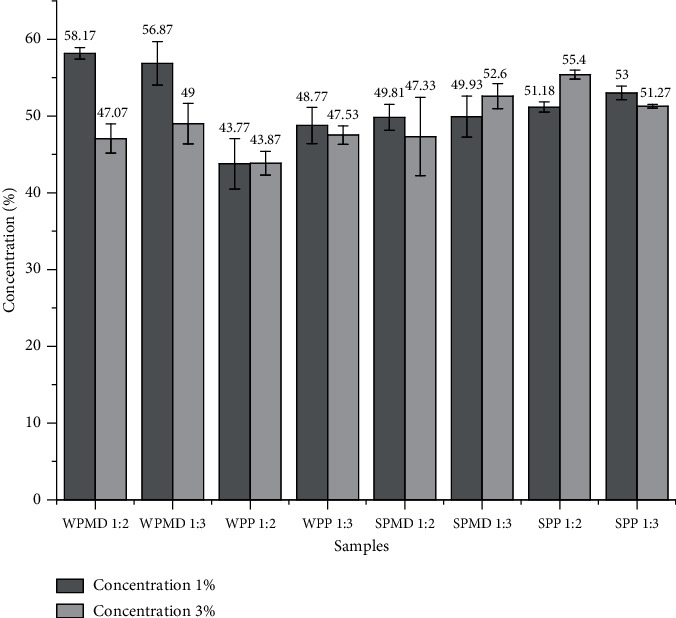
Surface tension of protein-polysaccharide conjugate solution with concentrations of 1.0% and 3.0% (surface tension values are the mean of three replicate samples; different letters indicate significance in the mean surface tension value at the 95% level).

The result shows that soy protein-maltodextrin (SPMD) conjugates lower the surface tension more than whey protein-maltodextrin (WPMD) conjugates at the same concentration (Figure 2). The surface tension of the soy protein conjugates differed significantly from that of the whey protein. The order of the surface tension is SPMD 1 : 2 ≈ SPMD 1 : 3 < WPMD 1 : 2 ≈ WPMD 1 : 3. This phenomenon is similar to Kutzli et al. [51], who reported that the surface tension of the maltodextrin-SPI mixture was lower than the maltodextrin-whey protein isolate blend. Regarding Fang et al. [52], the whey protein isolate has lower surface activity than plant proteins, such as soy protein, pea protein, and rice protein. The closely packed globular structure of the whey protein slows the diffusion of proteins to the interface [53].

In contrast to more rigid whey proteins, soy protein has a mobile area structure that was believed to be responsible for a stronger unfolding at the interface and increased surface activity [54]. It can then be understood if the conjugates containing soy protein have a better ability to reduce surface tension than those containing whey protein. Another fact supporting this is that SPMD has higher protein levels than WPMD. The protein content of SPMD 1 : 2, SPMD 1 : 3, WPMD 1 : 2, and WPMD 1 : 3 was 11.89%, 10.98%, 4.08%, and 2.98%, respectively. According to Patino et al. [49], the properties of proteins and emulsifiers at the water-air interface depend on how these surface-active components are adsorbed or dispersed and their solubility in water. Therefore, it is likely that the presence of maltodextrin in the WPMD and SPMD conjugates does not determine the properties of these conjugates in reducing surface tension, because maltodextrin does not have active surface sites [31, 55]. The role of protein-linked polysaccharide in maintaining the stability of the emulsion is through the mechanism of adsorbing its hydrophilic sites on the surface of the droplet, thus increasing the thickness of the interfacial layer and with its steric repulsion force keeping the droplets separated [22]. It is also hypothesised that proteins are principally responsible for the change in surface tension of maltodextrin-protein blends [51].

#### 3.1.2. The Effect of Soy and Whey Protein in Conjugate Products Containing Pectin on Surface Tension

The conjugate products of pectin with soy or whey protein had a different surface tension phenomenon. The whey protein-pectin (WPP) conjugate was able to reduce surface tension more than the soy protein-pectin (SPP) conjugate. In contrast to maltodextrin-containing conjugates, the decrease in surface tension by pectin-containing conjugates did not appear to be influenced by protein. The protein contents of WPP 1 : 2, WPP 1 : 3, SPP 1 : 2, and SPP 1 : 3 were 5.18%, 4.31%, 11.49%, and 11.43%, respectively. Unlike maltodextrin, pectin shows a high negative charge density [56]. Surface properties come from a large number of acetyl groups and proteins [57], as well as amphiphilic parts and hydrophobic groups in pectin [58]. When proteins and hydrocolloids have a negative net charge, the positive charge in the protein will interact with the negative charge possessed by the polysaccharide to form a soluble complex [59]. These soluble formations can be adsorbed on the interface surface and function as an emulsifier. As stated by Patino et al. [49], the water solubility affects the properties of the emulsifier. Furthermore, it is believed that the disparity in molecular weight between WPP and SPP conjugates impacts the surface tension of both conjugates. Since soy protein is a high molecular weight emulsifier [60], the SPP conjugate is likely to have a higher molecular weight than the WPP conjugate. Moreover, Deng [60] asserted that emulsifiers characterised by low molecular weight have the potential to further decrease surface tension.

Consequently, it is reasonable to expect that WPP exhibits a lower surface tension compared to SPP. The difference in molecular weight in the types of polysaccharides and proteins affects the molecular weight of the conjugate produced, which then also affects the adsorption's rapidity. According to Zinoviadou et al. [61], the slow adsorption to the interface can be caused by the large size of the molecule. The ANOVA showed that the increase in the pectin ratio did not significantly affect the surface tension of the conjugate products. Conjugates with a protein-polysaccharide ratio 1 : 3 tended to have a higher surface tension than the 1 : 2 ratio. This phenomenon may be related to the unconjugated pectin dispersed in the water. Pectin has surface activity; therefore, in a homogeneous state in water, the surface area of pectin increases and can reduce surface tension. The decrease in surface tension is consistent with the increase in pectin concentration until an aggregation state forms, where surface tension cannot longer be reduced [62].

#### 3.1.3. The Effect of Concentration of Conjugate Products on Surface Tension

The results of the surface tension measurements (Figure 2) also provided information on the effect of the conjugate concentration on the surface tension of the conjugate samples. According to Perinelli et al. [63], as the concentration of emulsifier/surfactant increases, the surface tension will decrease. The adsorption of surfactant at the interface leads to a reduction in the repulsion of the interface due to the surfactant's attraction for both hydrophobic and hydrophilic phases. Consequently, a reduction in interfacial tension causes a change in interface, which is further induced by mechanical forces, resulting in the formation of an emulsion [64]. The higher the surfactant concentration, the more intense the reaction becomes, hence the increase in the decrease of surface tension. The increase in conjugate concentration in the emulsification process exhibited a general trend of reducing water surface tension, except for WPP 1 : 2, SPMD 1 : 3, and SPP 1 : 2 samples. However, the results of the ANOVA test indicate that all three samples with a concentration of 3.0% were not significantly different from the same sample at a concentration of 1.0%. The increase in surface tension in both conjugate samples was likely due to the saturation of the oil/water interface emulsifier or the attainment of limitable surface tension (limiting surface tension), as expressed by Burlatsky et al. [65]. In bulk concentration, surfactants aggregate to form micelles, called critical micellisation concentration (CMC). At this condition, the surfactant cannot anymore reduce surface tension [62]. The protein-polysaccharide conjugates produced from this study have the potential to be further developed and studied as natural emulsifiers because they have the potential to reduce the surface tension of water, especially WPP 1 : 2. The surface tension value of WPP 1 : 2 (43.77 dyne/cm^2^) is close to the surface tension of the commercial emulsifiers Tween 60 and Tween 80 which is around 40 dyne/cm^2^ at a concentration of 1% [66].

### 3.2. Emulsifying Index Activity

**Figure 3:**
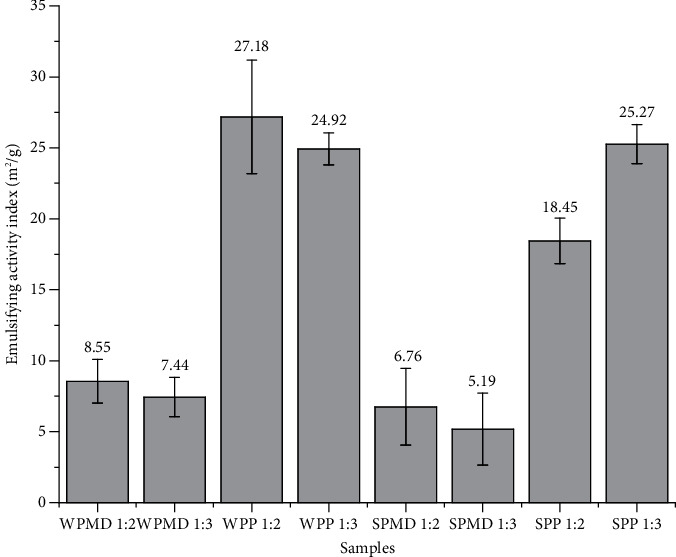
Emulsifying activity index of conjugates (EAI values are the mean of 3 replicate samples and three measurements; different letters indicate significance in the mean EAI value at the 95% level).

Emulsifying index activity (EAI) is an important parameter that determines the quality of an emulsifier. The test result showed that the conjugates formed containing pectin (WPP and SPP) had a more notable EAI than those using maltodextrin (WPMD and SPMD). The EAI values of WPP 1 : 2, WPP 1 : 3, SPP 1 : 2, and SPP 1 : 3 were 27.18, 24.92, 18.45, and 25.27 m^2^/g, respectively. The WPMD 1 : 2, WPMD 1 : 3, SPMD 1 : 2, and SPMD 1 : 3 have EAI values of 8.95, 7.44, 6.76, and 5.19 m^2^/g, respectively. The results of the variance analysis and Tukey's additional test showed that the EAI value of the WPP conjugate sample was significantly different from WPMD (*p* < 0.05), as well as between SPP and SPMD (Figure 3). In the oil-in-water emulsion system, the hydrophobic and hydrophilic parts of protein molecules are adsorbed at the interface of oil and water to form an amphipathic film. At the same time, the presence of polysaccharides has the role of thickening the film layer around the oil droplet. This condition prevents the flocculation and coalescence of protein molecules, increasing the stability of protein-emulsifying properties [67]. Maltodextrin and pectin have different structures that affect the character of adsorption. Maltodextrins have no surface activity, thus allowing them to stabilise the emulsion by gel formation or modification of the viscosity of the aqueous phase. On the other hand, pectin is a polysaccharide that has interfacial activity. Pectin contains protein residues and acetyl groups (4-5%), which help improve its emulsifying ability due to its hydrophobic nature [68]. Hydrophobic sites or chains adsorb on the surface of the oil-water interface. This action prevents flocculation and coalescence through electrostatic power and steric repulsion [14]. This assertion is consistent with the research conducted by Shu et al. [69], which concluded a positive correlation between the length of polysaccharide chain and the emulsification activity of the conjugates. Furthermore, Hernández-García et al. [70] revealed that the higher molecular weight could affect the increase in emulsifying ability. Polysaccharides possessing greater molecular weights tend to be more advantageous for conjugation because of their increased steric hindrance. The property confers advantages in reducing coalescence and enhancing the emulsifying properties of conjugates [71, 72].

As mentioned above, the molecular weight of pectin tends to be higher than that of maltodextrin, and, understandably, protein conjugation with pectin resulted in a higher emulsifying activity index than in conjugation with maltodextrin.

Figure 3 also shows the effect of different types of protein on the same kind of polysaccharide. Conjugate samples containing whey protein (WPMD and WPP) appear to have higher EAI values than conjugate samples containing soy protein (SPMD and SPP). However, the EAI value of WPMD was not significantly different from SPMD, nor was the EAI value of WPP significantly different from SPP except for SPP 1 : 2 (Figure 3). Based on the overall measurement results, it was found that the WPP 1 : 2 had the highest EAI value and was close to the EAI value of Tween 80 as a commercial emulsifier (graph not shown). This result is in agreement with surface tension measurements, where the WPP 1 : 2 conjugate sample performs best in reducing surface tension. According to Li et al. [73], emulsification activity is influenced by the ability of proteins to reduce interfacial tension.

### 3.3. Droplet Size, Polydispersity Index, and Zeta Potential

#### 3.3.1. Droplet Size

**Figure 4:**
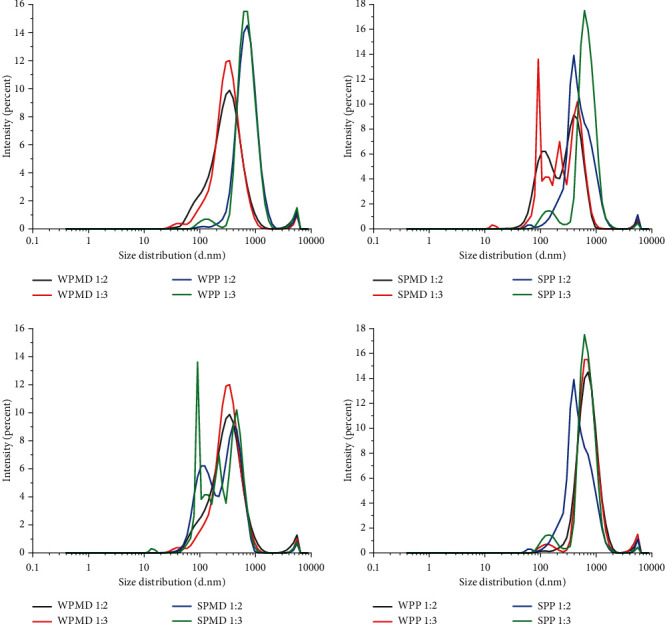
Size distribution by intensity of eugenol emulsion using various protein-polysaccharide conjugates resulting from DLS measurement. Comparison between conjugates with the same protein type (WPMD-WPP, SPMD-SPP) and conjugates with the same polysaccharide type (WPMD-SPMD, WPP-SPP).

The results showed that the WPMD emulsion samples had smaller droplets than other conjugate emulsions (Table 1), while the SPP emulsion had higher droplets. The droplet size distribution graph can be seen in Figure 4. All emulsions show a multimodal distribution, but each has distinct peaks. The eugenol emulsion with WPP conjugates appeared to have a larger particle size with higher intensity compared to that of the emulsion using WPMD conjugates. Figure 4 also shows that conjugates with a higher polysaccharide ratio affect the size of the droplets and the intensity of the particle/droplet size. Emulsions of WPMD 1 : 3 had a higher droplet size and intensity than emulsions of WPMD 1 : 2. The emulsions of WPP 1 : 3 and WPP 1 : 2 showed a similar phenomenon.

Protein types affect particle size, especially in particle size distribution. The SPMD 1 : 2 emulsion had a smaller droplet size (230 nm) than the emulsion with WPMD 1 : 2 (256 nm). The droplet size of the SPMD 1 : 3 emulsion seemed to be larger than that of the WPMD 1 : 3 emulsion at greater maltodextrin concentrations. However, statistical analysis indicated that this difference was not statistically significant. It should be noted that SPMD emulsions have more multimodal particle size distribution peaks than WPMD (Figure 4). The multimodal particle size distribution profile indicates the inhomogeneity of the emulsion. The difference in polysaccharide ratio in conjugates containing soy protein also tended to increase the droplet size. The droplet size of the SPMD 1 : 3 emulsion was higher than the SPMD 1 : 2 emulsion, as well as between the SPP 1 : 3 and SPP 1 : 2 emulsions, as indicated in Table 1. Figure 4 shows the droplet size graph of SPMD and SPP emulsions, highlighting the discernible disparity in the droplet size distribution between the two. When the multimodal distribution graph of both SPMD and SPP emulsions is examined, it becomes evident that the SPMD emulsion exhibits more peaks than the SPP emulsion.

The molecular weight of the conjugate product affects the droplet size of the resulting emulsion. Low molecular weight surfactants are more effective in producing emulsions with small droplet [14]. Nagaraju et al. [55] revealed that low molecular weight causes sodium caseinate to have rapid adsorbing properties at the interface and contributes to the formation of small particle. Considering that the molecular weight of pectin is greater than that of the maltodextrin [55, 74], it is reasonable to infer that the droplet size of WMPD emulsion is comparatively smaller than that of WPP. Similarly, the droplet size of the SPMD emulsion is smaller than that of the SPP emulsion. However, the adsorption properties of the polysaccharide conjugated with the protein also affect the particle size of an emulsion. Protein molecules or polysaccharides that are not entirely conjugated can also cause the large droplet size of the resulting emulsion. Free protein molecules may still have the function of emulsifying oil because they have surface-active or hydrophobic parts. However, even excess amounts can cause droplet accumulation [75]. Unabsorbed polysaccharides can lead to flocculation [76], resulting in large droplet, a wide particle size distribution, and instability of the emulsion.

#### 3.3.2. Polydispersity Index

The polydispersity index (PdI) is a value that indicates particle size heterogeneity. PdI values are expressed between 0 and 1, where 0 indicates a monodisperse particle dispersion system, while 1 indicates a highly polydisperse system [77]. The conjugate emulsions observed in this study had PdI values between 0.3 and 0.64, indicating an emulsion system that tends to be polydisperse. The analysis of emulsion droplet size measurements revealed that the whey protein conjugate emulsions (WPMD and WPP) exhibited smaller polydispersity index (PdI) values compared to those of the soy protein conjugate emulsions (SPMD and SPP). The probable reason for this phenomenon is that soy protein has a greater molecular weight than whey protein. Based on the result of Nagaraju et al. [55], PdI values can be influenced by the molecular weight of the carrier material in the formation of the particle multilayer system. However, the difference in PdI values was not significantly different, except between WPP 1 : 2 and SPMD 1 : 3. The WPP 1 : 2 emulsion has the smallest PdI value (0.37), while SPMD 1 : 3 has the most significant PdI value (0.64). Figure 4 displays the graphical representation of the distribution of droplet sizes.

The SPMD 1 : 3 emulsion has a larger droplet size distribution with many peaks than the WPP 1 : 2 emulsion. The unimodal droplet size distribution pattern and low PdI value indicate high adsorbing or emulsifying properties at the interface [55]. Although the WPP 1 : 2 conjugate emulsion had a larger particle size of 651 nm compared to the WPMD 1 : 2 conjugate emulsion with a particle size of 256.5 nm, it should be noted that the former showed a smaller polydispersity index (PdI). The constituent polysaccharide elements in the conjugate likely cause this phenomenon. Pectin has hydrophobic surface-active areas, making unconjugated pectin capable of adsorbing oil. The independent role of pectin is believed to help form an emulsion system that is relatively more homogeneous than that of emulsions with other types of conjugates. The conjugate product obtained through the Maillard reaction is a complex mixture that includes conjugated compounds and unconjugated proteins and polysaccharides [72].

The addition of pectin concentration to the whey protein-pectin conjugate (WPP 1 : 3) increased the PdI value. Djuardi et al. [78] have reported that variations in the concentration of protein-conjugated components can influence adsorption activity, leading to coalescence and the emergence of a diverse range of particle sizes. Another possible cause of multimodal distribution or high polydispersity is the aggregation of droplets due to reassociation and rearrangement of emulsifying materials or other materials involved in the emulsion [79]. The PdI values observed in the study showed a positive correlation with the emulsifying activity, as indicated by the emulsifying activity index (EAI). The findings indicated that the WPP 1 : 2 conjugate significantly exhibited the highest emulsifying activity, while SPMD 1 : 3 demonstrated the lowest value of the emulsifying activity index (EAI) (Figure 3).

#### 3.3.3. Zeta Potential

The electric charge interface influences the emulsion stability. The *ζ*-potential serves as an indicator of the level of interaction between emulsion droplets. The magnitude of the *ζ*-potential directly correlates with the strength of the repulsive force between the droplets, therefore influencing the stability of the system [80]. An elevated zeta potential corresponds to a strong electrostatic repulsion among particles, resulting in increased resistance to aggregation and a stronger tendency to remain distinct [14, 81, 82]. On the contrary, if the zeta potential exhibits a low value, the particles approach one another, aggregate, or flocculate, leading to an increase in the observed particle size [14].

The zeta potential values of all emulsion samples, as presented in Table 1, exhibit a negative value. Most emulsion samples have zeta potential values close to -30 mV, indicating the stability of the emulsion. WPP conjugates had higher zeta potential values (-37 mV and -34 mV) than WPMD (-24 mV and -26 mV). The zeta potential values of the WPP conjugates were comparatively higher (-37 mV and -34 mV) compared to WPMD (-24 mV and -26 mV). Although the droplet size of the WPMD emulsion was lower than that of WPP, the zeta potential measurements indicate that WPP exhibits more stability than WPMD. Emulsions are considered to exhibit stability when their zeta potential value is less than -30 mV or more than +30 mV [83]. The difference in zeta potential between WPP and WPMD was probably due to differences in the adsorption properties and surface activity of the two polysaccharides. The concentration of WPMD conjugate used in emulsification is likely insufficient to adsorb the oil entirely and reduce surface tension. This possibility is also evident from the fact that the surface tension of WPMD is higher than WPP's (Figure 2). Zhang et al. [75] reported that insufficiency of emulsifiers leads to lack of repulsion and increased coalescences, resulting in an unstable emulsion system. WPP conjugates may contain unconjugated pectin [72], enabling direct interaction with oil. Pectin is a polysaccharide that has surface activity and a nonpolar region. As a result, it functions to stabilise emulsions through the adsorption process on the surface of oil particles [84].

The zeta potential value of the soy protein-maltodextrin conjugate emulsion (SPMD) tends to be higher than the soy protein-pectin conjugate emulsion (SPP), but the difference was insignificant. This phenomenon is likely due to differences in the properties of maltodextrin and pectin, their interaction with soy protein, and their association with unconjugated proteins and polysaccharides. Soy protein has some surface-active globular proteins, as does pectin, although in smaller amounts. Both unconjugated soy protein and pectin are believed to influence the decrease in the zeta potential. According to Schröder et al. [85], the reduction in zeta potential due to the addition of protein is a result of the formation of dense multilayers that increase surface coverage. The multilayer formation can be derived from unconjugated soy protein or pectin containing protein. In addition, unconjugated pectin can also play a role in reducing zeta potential because it is related to the presence of carboxylic acid groups [84].

Increasing the concentration of polysaccharides in protein-polysaccharide conjugates decreases the zeta potential value in conjugates containing whey protein and increases the zeta potential value in conjugates containing soy protein; however, the change was not significant (*p* < 0.05). The difference in zeta potential trend depends on the type of protein. This phenomenon may be due to the kind of protein and polysaccharide and the presence of unconjugated proteins and polysaccharide molecules. The whey protein used in the study has a low protein content of 11%, allowing unconjugated maltodextrin and pectin to occur in the conjugation process. The existence of biopolymers on the surface of oil droplets could lead to the formation of a viscoelastic layer, which hinders the coalescence process and consequently improves the stability of the emulsions [86]. However, an excessive concentration leads to an electrostatic screening phenomenon, decreasing the charge of the droplet and the zeta potential [87]. The soy protein has a high protein content of 81.06%. Increasing the concentration of maltodextrin or pectin in the conjugation process with soy protein might increase the production of conjugates. In addition, there may also be unconjugated protein or free protein. This was confirmed by SEM observations of spherical and smooth-surfaced particles that were not found in the whey protein-containing conjugate samples. The free protein can be adsorbed on oil droplets because it has a surface-active or hydrophobic side. The quantity of protein affects the formation of a viscoelastic film around the oil droplet and the stability of the emulsion [88, 89].

### 3.4. Differential Scanning Calorimetry (DSC) of Conjugates

Emulsions, especially those stabilised with proteins, are very sensitive to heat [90]. Thermal stability is one of the properties that must be possessed by food emulsifiers, because food processing generally uses heat processes. DSC is a sensitive tool for assessing the thermal stability and conformation of proteins [91, 92]. Protein-containing emulsifiers are prone to denaturation during heat processing. The denaturation of globular proteins is related to the destruction of hydrogen bonds and hydrophobic interactions that strengthen and stabilise the conformation of the native protein [56]. The process is followed by interactions between proteins and aggregation between protein molecules that coat the oil in an emulsion system [93]. Aggregation can be physically limited by the presence of conjugated proteins at the oil/water interface with a function of steric stabilisation [93]. In addition, the presence of polysaccharides as part of the protein-polysaccharide conjugate can reduce the solvent availability of proteins through the crowding effect and act as steric spacers between protein molecules, protecting globular proteins from aggregation and remaining in a steady-state structure [56, 94]. This mechanism is thought to occur in WPMD conjugates, giving WPMD certain thermal properties.

**Figure 5:**
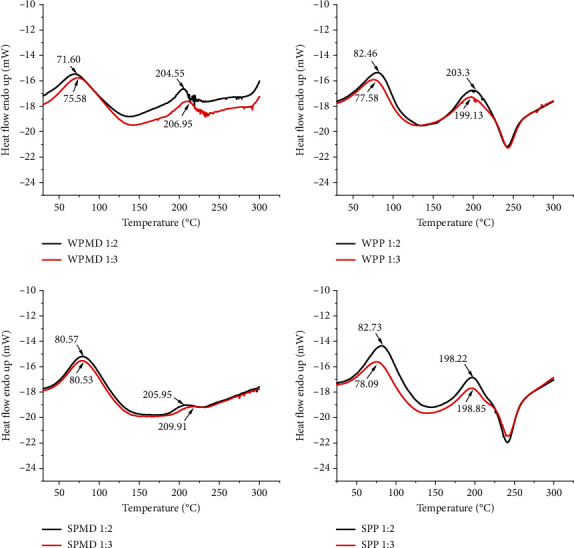
DSC thermogram of conjugate samples.

WPMD conjugates had the peak temperature at 71.60°C, which is probably a molecular structural change and at 204.55°C (Figure 5), which is related to the decomposition [55]. The decomposition temperature of both WPMD 1 : 2 and WPMD 1 : 3 was higher than that of whey protein (150°C) and maltodextrin, presumably related to its conjugated form with maltodextrin. The Maillard reaction can reduce the sensitivity of proteins to heat treatment by inhibiting intermolecular interactions between protein molecules and maintaining the native structure of the protein [94]. The results of this study are in line with the previous research reporting that the thermal stability of whey protein isolates increased through glucose conjugation [95]. The increase in thermal stability is also indicated by an increase in the value of Δ*H*. The Δ*H* value of WPMD conjugate is higher than that of the whey protein. According to Ibanoglu [56], an increase in enthalpy change may occur due to a decrease in protein aggregation due to denaturation.

Soy protein-maltodextrin (SPMD) had a higher peak temperature and Δ*H* value than soy protein, indicating higher thermal stability. Compared to WPMD, the conjugate SPMD also showed higher values Δ*H*. This is related to the difference in protein composition between the two types of protein. The peak temperature of the soy protein (82.50°C) was lower than the whey protein; however, the soy protein had a higher Δ*H* value. The difference in heat stability between soy protein and whey protein is related to the denaturation behaviour and structural complexity of soy protein [96]. Lower Δ*H* value indicates that less energy is required for denaturation [56]. Thus, SPMD can be said to have better thermal stability than WPMD because it requires more energy for denaturation. In conjugate samples containing soy protein, the ratio of maltodextrin to pectin mass did not affect the endothermic peak temperature. Meanwhile, increasing the mass ratio of maltodextrin in conjugation with whey protein slightly increased the endothermic peak temperature but not with an increase in the pectin ratio. Conjugates containing maltodextrin (WPMD and SPMD) tend to have higher Δ*H* values than their native proteins. In addition to that, conjugates containing pectin tend to have a higher enthalpy change value than those with maltodextrin.

**Figure 6:**
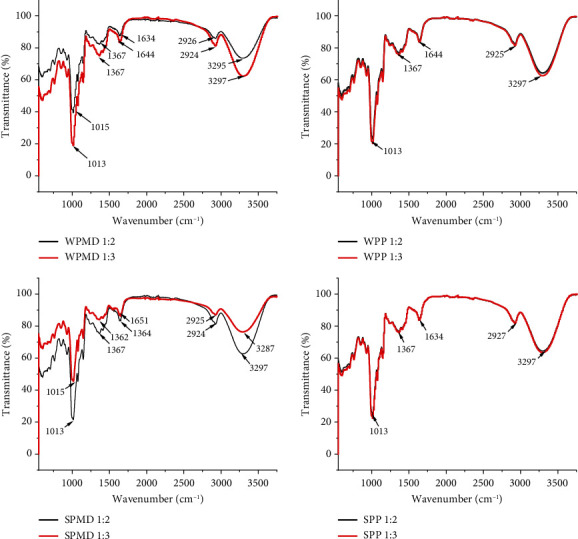
Infrared spectra of conjugate samples.

**Figure 7:**
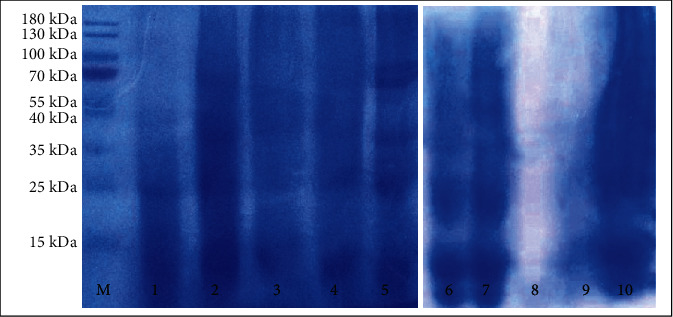
SDS-PAGE pattern of conjugate samples (M: marker; 1: SPMD 1 : 3; 2: SPMD 1 : 2; 3: SPP 1 : 3; 4: SPP 1 : 2; 5: native soy protein; 6: WPMD 1 : 3; 7: WPMD 1 : 2; 8: WPP 1 : 3; 9: WPP 1 : 2; 10: native whey protein; WPMD: whey protein-maltodextrin; WPP: whey protein-pectin; SPMD: soy protein-maltodextrin; SPP: soy protein-pectin).

**Figure 8:**
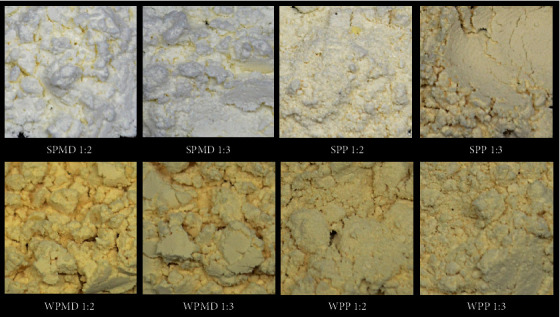
Visual appearance of conjugate spray-dried powder.

**Figure 9:**
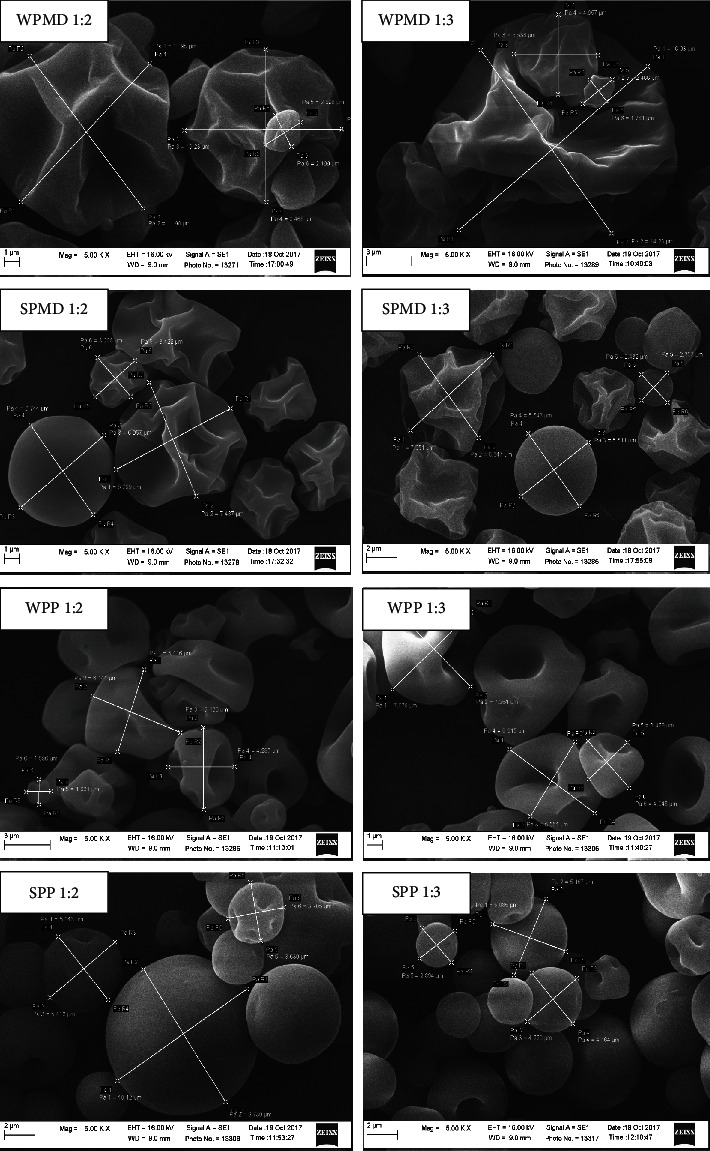
Surface morphology of conjugate spray-dried powder at a magnification of 5000 times. WPMD: whey protein-maltodextrin; WPP: whey protein-pectin; SPMD: soy protein-maltodextrin; SPP: soy protein-pectin.

Different types of polysaccharides provide other thermal characteristics. This fact can be seen from the enthalpy value generated from the scanning calorimetry results in the WPMD and WPP samples and between SPMD and SPP. Data on the enthalpy value of all conjugates formed can be seen in Table 2 and DSC images with enthalpy values are in the supplementary data in Figures 1-12. Unlike maltodextrin, pectin has a negative charge. When proteins and hydrocolloids are negatively charged, the attraction of the positive charge in the protein and the negative charge in the polysaccharide will result in a soluble protein-polysaccharide complex [59]. The increased negative charge also strengthens the electrostatic repulsion between proteins, preventing protein aggregation [97]. This is likely the reason for the higher values of Δ*H* WPP and SPP compared to WPMD and SPMD. The conjugate sample containing pectin showed an enthalpy value lower than that of the native protein. Ibanoglu [56] stated that the lower Δ*H* value of conjugates indicates less energy for denaturation; it is likely related to partial denaturation before heat treatment due to the high charge density of biopolymers.

### 3.5. Fourier Transform Infrared (FTIR) of Conjugates

Fourier transform infrared (FTIR) analysis was performed in order to characterise the bonds present in the samples. Each functional group possesses a specific absorption region. This approach enables the identification of alterations or the development of new chemical bonds. The bonds that form amide groups (C-O, N-H, and C-N) associated with the Maillard reaction products are absorbed at wavelengths of 800-1800 cm^−1^ [98]. The IR spectra of the conjugate samples are depicted in Figure 6, while the IR spectra of whey protein, soy protein, maltodextrin, and pectin are contained in the supplemental data. The featured bands at 1644 cm^−1^ in the conjugate samples containing whey protein (WPMD and WPP) are called C-O stretching of amide I. The absorption area of amide is divided into two parts: amide I between 1600 and 1700 cm^−1^ and amide II at wavelengths around 1550 cm^−1^ [99]. Compared with native whey protein spectra, the amide I group in the conjugate sample experienced a shift in wavelength and decreased absorption intensity. The whey protein had an amide I group at 1634 cm^−1^ with an absorption intensity of 25% and then shifted to 1644 cm^−1^ in conjugate WPMD with an absorption intensity of about 15%. The band at 1645 cm^−1^ was related to the Schiff base's C=N stretching vibration, which resulted from the Maillard reaction [100].

The whey protein spectra also showed the presence of an amide II group at 1538 cm^−1^. However, this band was not detected in the conjugate sample. According to Liu et al. [95], changes and decreases in intensity in the conjugate product occur due to vibrations in the amide I and amide II groups due to the deformation of the N-H bond and the stretching of the C-N bond. In addition, OH groups from maltodextrin and amino groups from whey protein are consumed during heating in the Maillard process. This event also explained why amide II (N-H) groups were not detected in the conjugate product. These results were also supported by the results of Su et al. [101], who found a gradual decrease in intensity at a wavelength of 1600-1400 cm^−1^ during the Maillard reaction between carboxymethylcellulose (CMC) and soy protein isolates. Previous studies have explained that functional groups, including NH_2_, especially lysine, may be lost [102].

The decrease in the intensity of amide I and amide II is usually followed by the formation or increase of amide III groups arising mainly from the stretching of C-N (pyrazine and Schiff base), which is the result of the Maillard reaction [103] and the deformation of the N-H, in the wavelength range of 1240-1450 cm^−1^. The original whey proteins had bands at 1260, 1358, and 1422 cm^−1^, representing the amide III group (Figure 6). The conjugation process caused a band shift, and the conjugate samples had bands between 1367 and 1368 cm^−1^. The transmittance percentage of WPMD 1 : 3 was relatively smaller than that of WPMD 1 : 2, while WPP 1 : 3 and WPP 1 : 2 had a similar transmittance percentage. In conjugation with whey protein, an increase in maltodextrin mass tended to increase absorption intensity, indicating an increase in C=O bond absorption (amide I).

The opposite phenomenon occurred in the conjugation of maltodextrin with soy protein. The increase in maltodextrin mass in the ratio between polysaccharide and protein caused a change in the wavelength of amide group I, which was initially 1634 cm^−1^ in the soy protein to 1651 cm^−1^ in the soy maltodextrin (SPMD) protein conjugate 1 : 3. The SPMD 1 : 2 conjugate had the same amide I group wavelength as soy protein. Increasing the mass of pectin in conjunction with whey protein and soy protein does not affect the absorption intensity. The analysis showed that the conjugates WPP 1 : 2 and WPP 1 : 3 have coincident spectra and SPP 1 : 2 and SPP 1 : 3.

### 3.6. SDS-PAGE

SDS-PAGE analysis was carried out to confirm the covalent bonds between proteins and polysaccharides. In the lane of SPMD 1 : 3, the protein bands were seen at molecular weights of 25 kDa and then appeared to fade to more than 180 kDa. The waning intensity of protein bands in the conjugate sample indicates the occurrence of conjugation between protein and polysaccharides during the Maillard reaction [104–106]. SPMD 1 : 2 seemed to show a deeper intensity of colour than the SPMD 1 : 3 conjugates (Figure 7). Furthermore, the SPMD 1 : 2 line showed scattered bands in the range of 70 kDa to a molecular weight of 180 kDa, indicating the conjugation of soy protein and maltodextrin. This statement is based on the results of a similar study [107]. The decrease in blue colour intensity indicates the conjugation between soy protein and dextran through the Maillard reaction [108]. Another finding stated that the bands fade out, suggesting that not all proteins interact with polysaccharides and form conjugates [19].

There was almost no difference in the SDS-PAGE pattern between SPP 1 : 2 and SPP 1 : 3. The intensity of the bands identified in the soy protein appears to fade. Faintly visible bands of typical proteins at molecular weights of 15 kDa and 25 kDa then diminished and formed polydispersed bands until the top of the separating gel, indicating the formation of the higher molecular weight of the product [109]. According to Ma et al. [110], the disappearance of protein bands in the conjugate product may be related to the large number of grafted peptides in the conjugate. Both WPMD conjugates, 1 : 2 and 1 : 3, also showed similar SDS-PAGE patterns.

The native whey protein bands were clearly seen in molecular weights of 15 kDa and 25 kDa. This pattern is not much different from the whey protein isolates that have patterns at 14 kDa (*α*-lactalbumin), 18 kDa (*β*-lactoglobulin), 37 kDa (*β*-lactoglobulin dimer), and 67 kDa (bovine serum albumin) [47]. The polydispersion of bands occurred and faded at molecular weights of more than 180 kDa. No staining was seen in WPP 1 : 3 (lane 8), while WPP 1 : 2 (lane 9) showed a broadband with a low stain intensity between 10 and 35 kDa molecular weight. Each conjugate type has different intensity bands, possibly due to transformation of the protein molecule that occurs later in the reaction [111].

There are several possible causes for this phenomenon. It was probably related to the low protein content of whey protein and the nature of the pectin that forms the gel, thus inhibiting protein separation in electrophoresis. Compared to WPP, the SPP conjugate lane showed more noticeable staining results. The band in lane SPP appeared diminished and diffused along the gel. This finding is the same as the results of Ma et al. [112]; bands of the soy protein isolate-pectin conjugate faded out in the range of molecular weights of 15 kDa, 25-35 kDa, and 55-70 kDa. Extensive and spread-out bands suggest the conjugation of protein with polysaccharides and the formation of molecules of high molecular weight [39].

### 3.7. Physical Appearance and Surface Morphology of Conjugate Spray-Dried Powder

#### 3.7.1. Physical Appearance

The difference in product colour observed in conjugate products containing whey protein and soy protein can be attributed to the different interactions between soy and whey protein with maltodextrin or pectin during the Maillard process (Figure 8). The Maillard reaction occurs through a series of three distinct stages. During the initial phase, a covalent linkage is established between the carbonyl group present on the reducing sugar of the polysaccharide and the amino group found on the amino acid, peptide, or protein. This interaction forms a Schiff base, producing an N-substituted glycosylamine compound. Subsequently, the glycosylamine undergoes rearrangement, creating a more stable Amadori product [108]. At this stage, no alterations were observed in the resultant product's colour, flavour, metal chelation, and toxicity.

The soy protein-maltodextrin (SPMD) conjugate had the palest colour among the other conjugate samples. This may be related to the time it takes for the Maillard reaction to occur. Boonlao et al. [105] found that the formation of Amadori products in the conjugation of soy protein isolate and maltodextrin occurred between day one and day three at 60°C, based on the measurement of the progress of the Maillard reaction. The conjugation reaction of soy protein-maltodextrin in this study took place at 90°C for 2 hours. Therefore, it is conceivable that the SPMD conjugate product derived from the Maillard reaction during the early or in the development stage of the Amadori product, resulting in the absence of any observable alteration in colour. Changes in the product's colour to become darker occur at the intermediate and final stages of the Maillard reaction [113, 114]. In addition to the Maillard reaction time, browning appears to be affected by the type of protein. Conjugates containing whey protein seemed browner than conjugates containing soy protein. This may be related to the kind of whey protein. The bovine milk whey protein used in the study has a high carbohydrate content (75.77%). Given the explanation that the more carbonyl groups will affect the browning reaction [115], it can be understood why the colours of WPMD and WPP are darker than those of SPMD and SPP.

Figure 8 shows that the differences in protein affect the colour of the resulting conjugate products. The colour difference may be related to the colour of the whey protein used in the conjugation process. Whey protein powder used in this research had a darker colour than soy protein powder. The colour of the resulting conjugate product may also indicate the occurrence of a Maillard reaction and the success of the conjugation process. Monitoring the progress of the Maillard reaction can be achieved by assessing many factors, including the advancement of colouration, changes in pH levels, and the production of insoluble reaction products [116]. The whey protein-maltodextrin (WPMD) conjugate powder appears browner in colour than the whey protein-pectin (WPP) conjugate powder. This is likely due to the molecular weight difference between maltodextrin and pectin. Several studies have shown that the variations in the molecular weight of polysaccharides during conjugation with proteins influence the degree of browning observed in the resulting conjugate products. Klinchongkon et al. [117] showed that the WPI-pectin conjugate sample with the lowest molecular weight has the highest increase in brown colour intensity. The lower the molecular weight of pectin, the higher the number of reducing end groups [118]. According to Spotti et al. [115], the number of carbonyl groups on polysaccharides can accelerate the occurrence of the Maillard reaction, which can increase the intensity of the brown colour. In addition, it is also known that polysaccharides rich in reducing sugars are more susceptible to browning during the Maillard reaction. Protein conjugation with long-chain polysaccharides exerts a suppressive effect on the appearance of enzyme browning [119]. The fact that maltodextrin has a molecular weight (17-20 kDa) [74], which is less than pectin (60-130 kDa) [55], may explain the above phenomenon. This mechanism also applies to the colour difference between SPMD and SPP.

The influence of protein and carbohydrate structure on the time of the Maillard reaction seems probable. Under identical times and conditions of the Maillard reaction, the resultant conjugate products may arise from distinct stages of the Maillard reaction (early, intermediate, or final stage), depending upon the specific protein and polysaccharide involved.

#### 3.7.2. Surface Morphology

The surface morphologies of the spray-dried powder samples were observed with scanning electron microscopy (SEM). A total of 8 conjugate samples were observed. Images were sought that depicted sample particles of various sizes to show the range of particle sizes. In addition, particles with different morphologies show any changes in the particles during the drying process of the conjugate samples. Captured images were taken six times at magnifications of 100, 200, 500, 1000, 3000, and 5000 times. This article only shows the image at a magnification of 5000 times (Figure 9).

Conjugate samples that contain maltodextrins, such as WPMD and SPMD, tended to have irregular shapes with curved and shrunken surfaces (Figure 9). The curves on the surface were formed from the shrinkage of the microcapsule during the drying process, followed by a cooling process. As reported earlier, the rapid loss of water and shrinkage of the particle surfaces during spray drying cause concave deformation and uneven surfaces of the powder samples [120]. The presence of sugars affects the surface irregularity of microcapsules [121]. This statement explains why the particles observed in the WPMD samples have more indentations and shrunken surfaces than those observed in the SPMD samples. WPMD contain sugar derived from maltodextrin and whey protein. Based on the approximate results of the analysis, the whey protein used in the study has a protein content of 7.85% and a carbohydrate content of 75.77%, while soy protein has a protein content of 81.06% and a carbohydrate content of 5.42% (data not shown). SPMD conjugates also have spherical particles with a smooth surface without indentation. This observation aligns with the research conducted by Shao et al. [120]; it is observed that the incorporation of the soy protein isolate affects the morphology, resulting in a more spherical particle form. The utilisation of different protein types affected the microstructure of the spray-dried powder conjugates. Similar particles were seen to dominate in the soy protein-pectin (SPP) conjugate samples, but not in the whey protein-pectin (WPP) conjugates.

A different phenomenon is also observed in the morphological characteristics of powder conjugates that incorporate pectin. The morphology of whey protein-pectin (WPP) conjugate particles resembles that of whey protein-maltodextrin (WPMD) particles; however, it has a reduced presence of indentations and wrinkles. Similar results were observed when soy protein-maltodextrin (SPMD) and soy protein-pectin (SPP) particles. In SPMD, particles with concave surfaces appear to dominate over SPPs. Hence, it may be revealed that maltodextrin exerts a more significant impact on developing indentations and wrinkles in spray-dried powder particles than whey protein. The morphological distinctions between WPP and SPP particles are evident. Most soy protein-pectin (SPP) particles in Figure 9 have a spherical morphology that is characterised by a smooth surface. In addition to the impact of the soy protein, the morphological characteristics of SPP were affected by pectin. With respect to Nagaraju et al. [55], the smooth surface may be attributed to robust cross-linked, elongated chain molecules. Similar results were also stated by Pereira et al. [122], who used pectin to form the microstructure.

The concentration of polysaccharides (maltodextrin and pectin) in the mass ratio between protein and polysaccharide did not appear to affect the morphology of the conjugate particles produced. Observations showed that there was no difference in particle shape between WPMD 1 : 2 and WPMD 1 : 3, as well as between SPMD 1 : 2 and SPMD 1 : 3, between WPP 1 : 2 and WPP 1 : 3, and between SPP 1 : 2 and SPP 1 : 3. The difference in particle shape is more influenced by the type of protein and polysaccharide.

## 4. Conclusions

The conjugation between plant protein (soy protein) and animal protein (whey protein) with non-surface-active polysaccharide (maltodextrin) and surface-active polysaccharide (pectin) properties has been well demonstrated. Protein-polysaccharide conjugation through the Maillard reaction has produced conjugate products with unique properties based on visual appearance and surface morphology. Increasing conjugate product concentration tends to decrease surface tension. Conjugates containing whey protein tended to lower surface tension than those containing soy protein. Whey protein-pectin conjugates with a mass ratio of 1 : 2 and a concentration of 1% exhibit the lowest surface tension at 43.77 dyne/cm^2^. The emulsifying activity index was highest in this conjugate sample at 27.18 m^2^/g.

The emulsions using whey protein-maltodextrin conjugates had droplet sizes smaller than those using other conjugate types (256.5 nm). However, the WPP showed better polydispersity index and zeta potential values, indicating the stability of the emulsion. The stability of conjugate products against high temperatures was demonstrated by conjugates containing maltodextrin. The WPMD and WPP conjugates clearly show the presence of C=N groups at the 1644-1645 cm^−1^ peaks, indicating the success of the conjugation process. The fading or disappearance of protein bands and the formation of polydispersed bands also prove the formation of protein-polysaccharide conjugates. Based on these test results, whey protein-pectin (WPP) and whey protein-maltodextrin (WPMD) have better emulsifying properties than soy protein-pectin (SPP) and soy protein-maltodextrin (SPMD). However, further research is needed to confirm and elaborate on the properties of whey protein-pectin and whey protein-maltodextrin conjugates that may support their ability as emulsifiers, including molecular weight, conjugation efficiency, conjugate yield, surface hydrophobicity, interfacial properties, and storage stability.

## Figures and Tables

**Table 1:** Droplet size, polydispersity index, and zeta potential of conjugates.

Protein-polysaccharide conjugates	Droplet size (nm)	Polydispersity index	Zeta potential (mV)
WPMD 1 : 2	256.5 ± 11.7 ab	0.49 ± 0.06 ab	−24.55 ± 0.35 ab
WPMD 1 : 3	293.5 ± 8.3 ab	0.48 ± 0.03 ab	−22.85 ± 0.35 a
WPP 1 : 2	651.5 ± 17.2 ab	0.37 ± 0.01 b	−37.20 ± 0.28 d
WPP 1 : 3	766.7 ± 43.6 a	0.44 ± 0.01 ab	−34.55 ± 3.32 cd
SPMD 1 : 2	230.2 ± 38.2 b	0.52 ± 0.09 ab	−30.25 ± 0.49 bc
SPMD 1 : 3	549.3 ± 344 ab	0.64 ± 0.09 a	−32.10 ± 3.11 cd
SPP 1 : 2	592.1 ± 0.0 ab	0.52 ± 0.05 ab	−29.80 ± 0.28 bc
SPP 1 : 3	752.7 ± 137.7 ab	0.55 ± 0.05 ab	−30.40 ± 1.56 bcd

Data represent the mean of three replicates. Different letters indicate significant differences among the samples (*p* < 0.05).

**Table 2:** Peak temperature and enthalpy change values of whey protein, soy protein, maltodextrin, pectin, and conjugate products.

Sample	Peak temperature (°C)	Enthalpy change/Δ*H* (J/g)
Whey protein	150	66.22
Soy protein	82.50	175.38
Maltodextrin	80.47	118.08
Pectin	77.05; 155.38; 208.43	192.09; 47.19; 193.95
Whey protein-maltodextrin 1 : 2	71.60; 204.55	116.80; 9.27
Whey protein-maltodextrin 1 : 3	75.58; 206.95	156.03; 12.44
Whey protein-pectin 1 : 2	82.46; 203.3	143.82; 187.87
Whey protein-pectin 1 : 3	77.58; 199.13	143.03; 210.03
Soy protein-maltodextrin 1 : 2	80.57; 205.95	187.79; 8.92
Soy protein-maltodextrin 1 : 3	80.53; 209.01	193.09; 7.98
Soy protein-pectin 1 : 2	82.73; 198.22	181.43; 181.00
Soy protein-pectin 1 : 3	78.09; 198.85	166.866; 145.30

## Data Availability

The data from this research have never been published anywhere, either partly or entirely. Requests for data sets used in the current study should be addressed to the corresponding author.

## Supplementary Materials

Supplementary Materials All figures of supplementary material can be found in the file Supplementary data of DSC and FTIR. Figure 1: DSC thermogram of whey protein. Figure 2: DSC thermogram of soy protein. Figure 3: DSC thermogram of maltodextrin. Figure 4: DSC thermogram of pectin. Figure 5: DSC thermogram of whey protein-maltodextrin conjugated with ratio 1 : 2 (WPMD 1 : 2). Figure 6: DSC thermogram of whey protein-maltodextrin conjugated with ratio 1 : 3 (WPMD 1 : 3). Figure 7: DSC thermogram of whey protein-pectin conjugated with ratio 1 : 2 (WPP1 : 2). Figure 8: DSC thermogram of whey protein-pectin conjugated with ratio 1 : 3 (WPP1 : 3). Figure 9: DSC thermogram of soy protein-maltodextrin conjugated with ratio 1 : 2 (SPMD1 : 2). Figure 10: DSC thermogram of soy protein-maltodextrin conjugated with ratio 1 : 3 (SPMD1 : 3). Figure 11: DSC thermogram of soy protein-pectin conjugated with ratio 1 : 2 (SPP1 : 2). Figure 12: DSC thermogram of soy protein-pectin conjugated with ratio 1 : 3 (SPP1 : 3). Figure 13: FTIR spectra of whey protein. Figure 14: FTIR spectra of soy protein. Figure 15: FTIR spectra of maltodextrin. Figure 16: FTIR spectra of pectin.
