# Supplementary material for: The Potency of Maillard Conjugates Containing Whey Protein as Natural Emulsifier
Source: Int J Food Sci. 2024 May 27;2024:3254132. doi: 10.1155/2024/3254132 (PMC11222009; doi:10.1155/2024/3254132)
Supplement: Supplementary Materials — All figures of supplementary material can be found in the file Supplementary data of DSC and FTIR. Figure 1: DSC thermogram of whey protein. Figure 2: DSC thermogram of soy protein. Figure 3: DSC thermogram of maltodextrin. Figure 4: DSC thermogram of pectin. Figure 5: DSC thermogram of whey protein-maltodextrin conjugated with ratio 1 : 2 (WPMD 1 : 2). Figure 6: DSC thermogram of whey protein-maltodextrin conjugated with ratio 1 : 3 (WPMD 1 : 3). Figure 7: DSC thermogram of whey protein-pectin conjugated with ratio 1 : 2 (WPP1 : 2). Figure 8: DSC thermogram of whey protein-pectin conjugated with ratio 1 : 3 (WPP1 : 3). Figure 9: DSC thermogram of soy protein-maltodextrin conjugated with ratio 1 : 2 (SPMD1 : 2). Figure 10: DSC thermogram of soy protein-maltodextrin conjugated with ratio 1 : 3 (SPMD1 : 3). Figure 11: DSC thermogram of soy protein-pectin conjugated with ratio 1 : 2 (SPP1 : 2). Figure 12: DSC thermogram of soy protein-pectin conjugated with ratio 1 : 3 (SPP1 : 3). Figure 13: FTIR spectra of whey protein. Figure 14: FTIR spectra of soy protein. Figure 15: FTIR spectra of maltodextrin. Figure 16: FTIR spectra of pectin. [file 3254132.f1.docx]

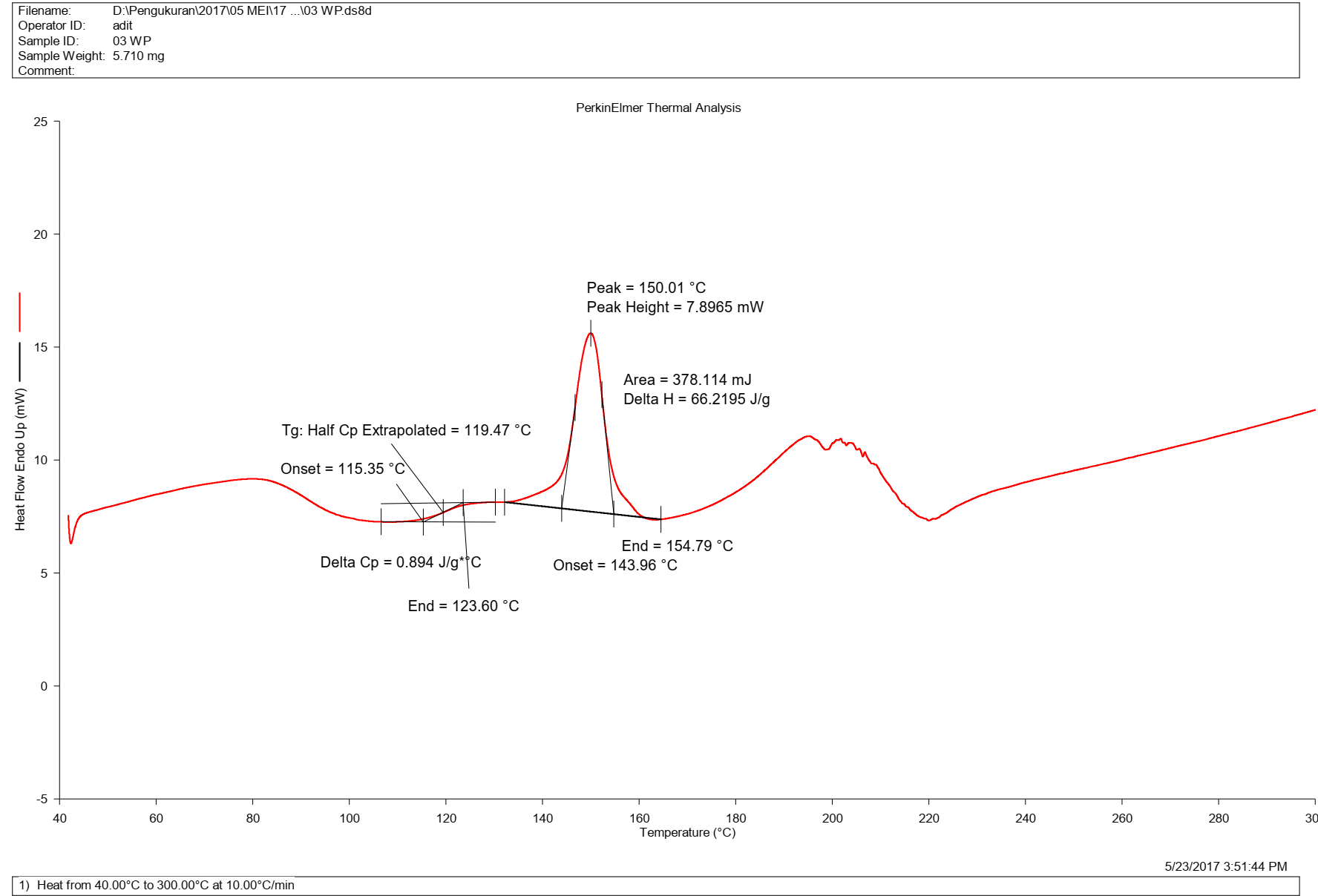


Figure 1. DSC thermogram of whey protein


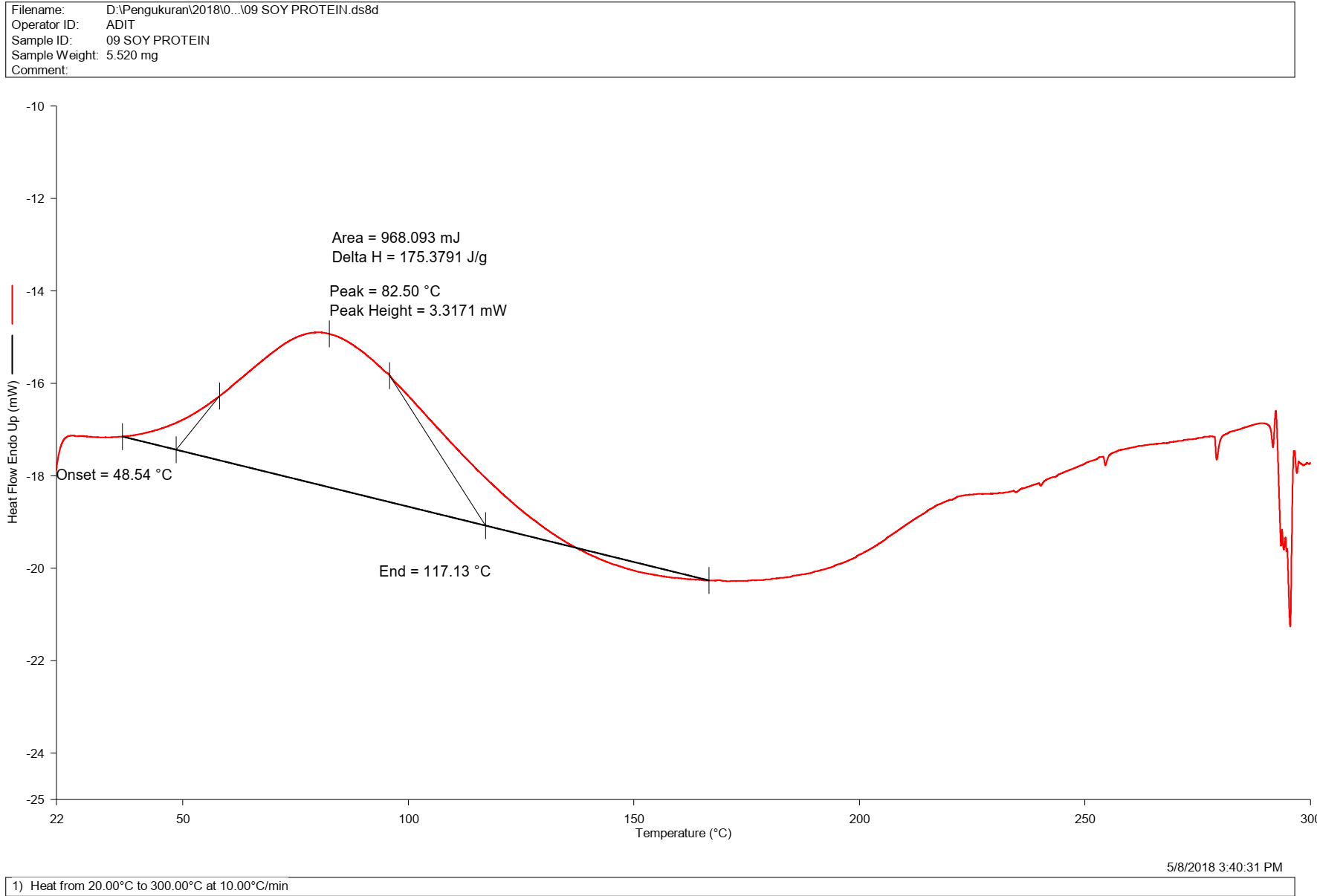


Figure 2. DSC thermogram of soy protein


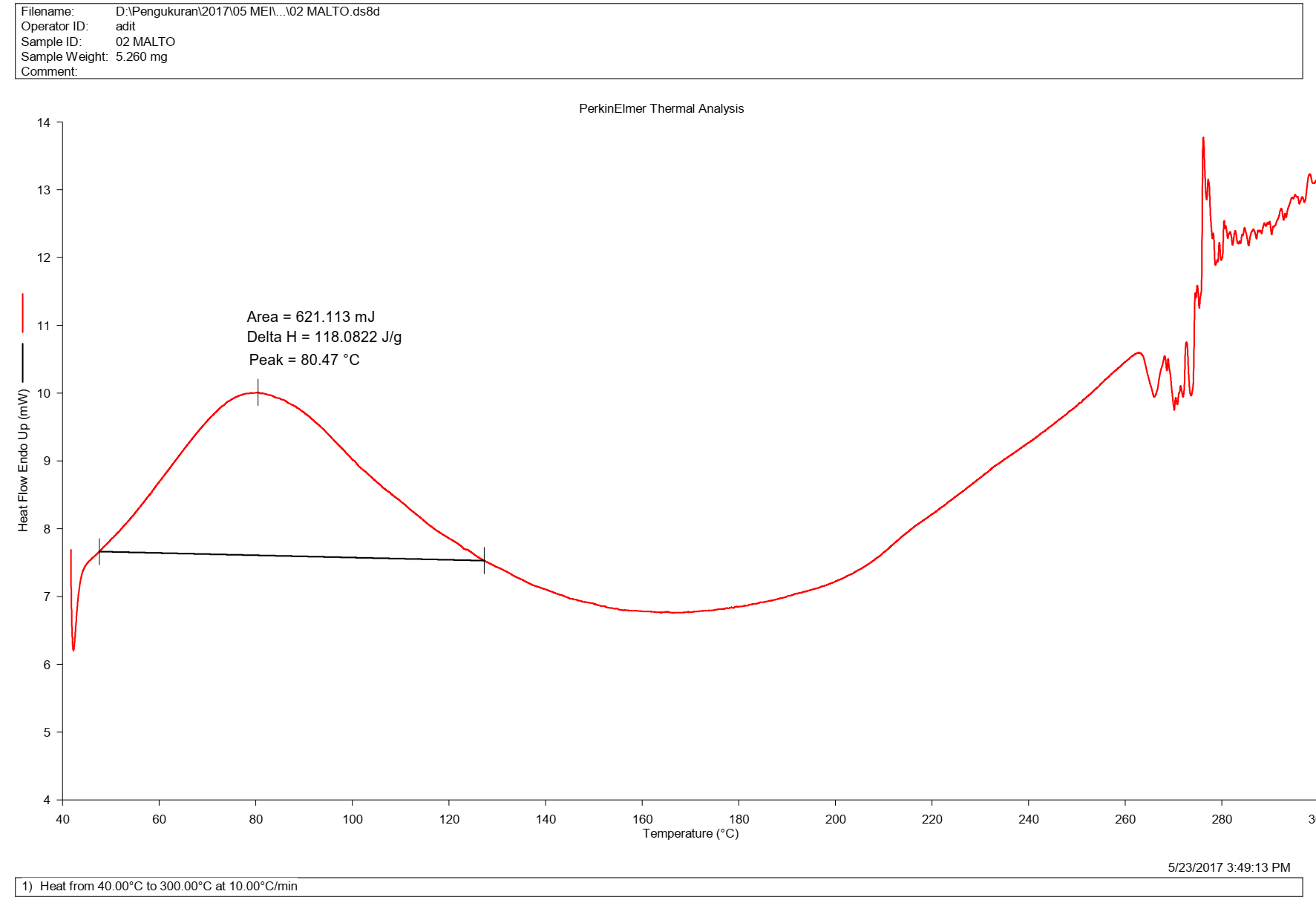


Figure 3. DSC thermogram of maltodextrin


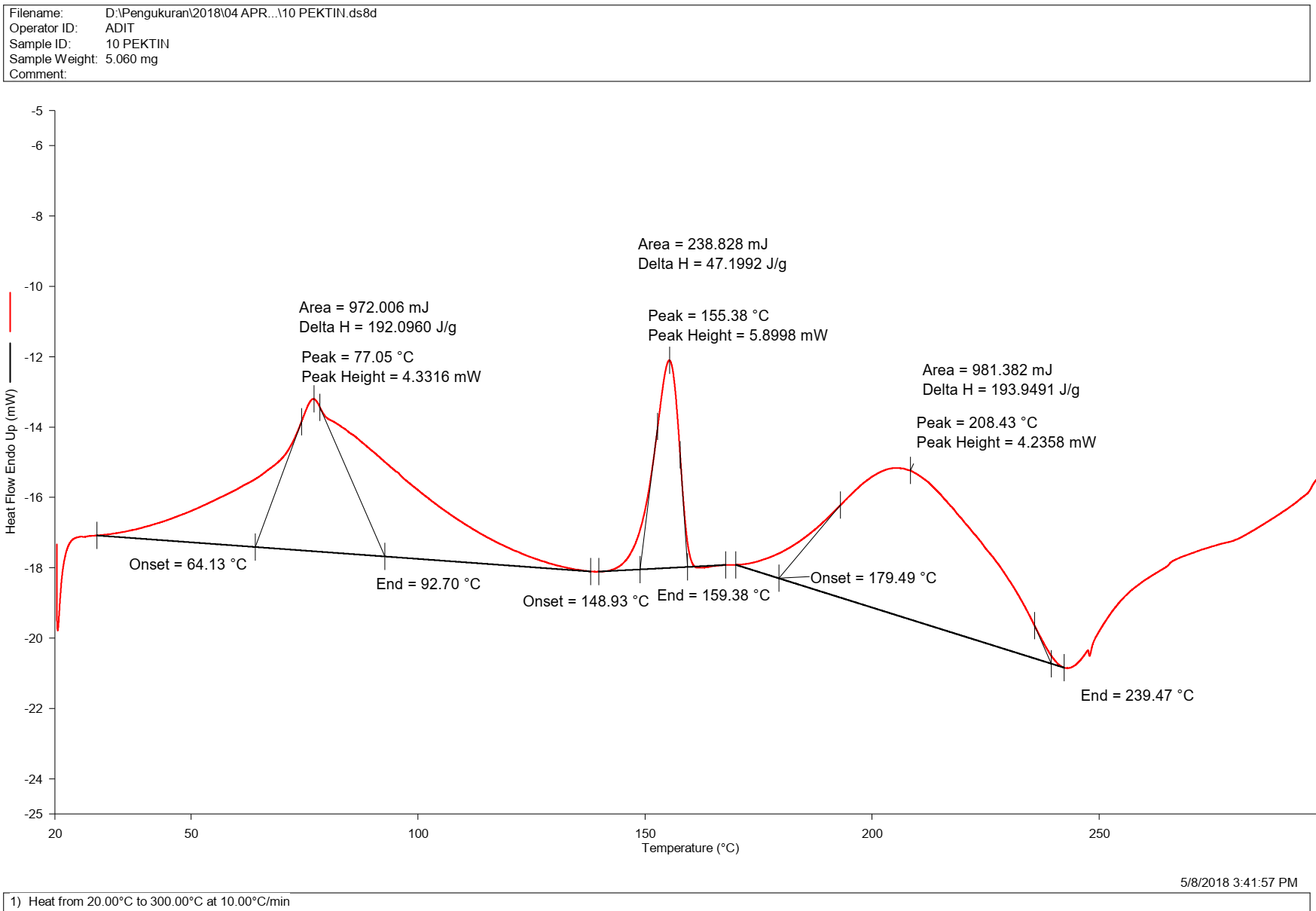


Figure 4. DSC thermogram of pectin


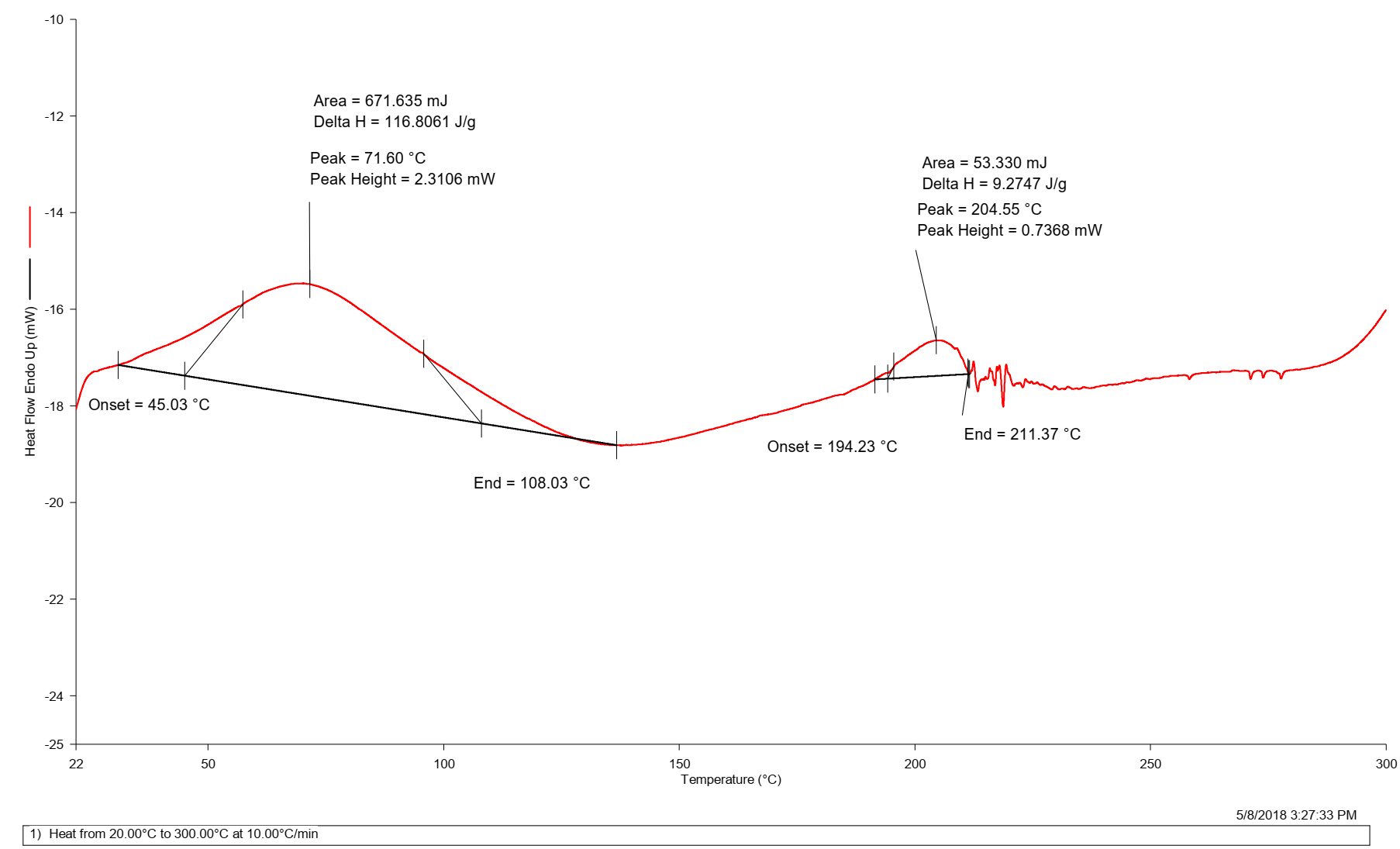


Figure 5. DSC thermogram of whey protein maltodextrin conjugated with ratio 1:2 (WPMD 1:2)


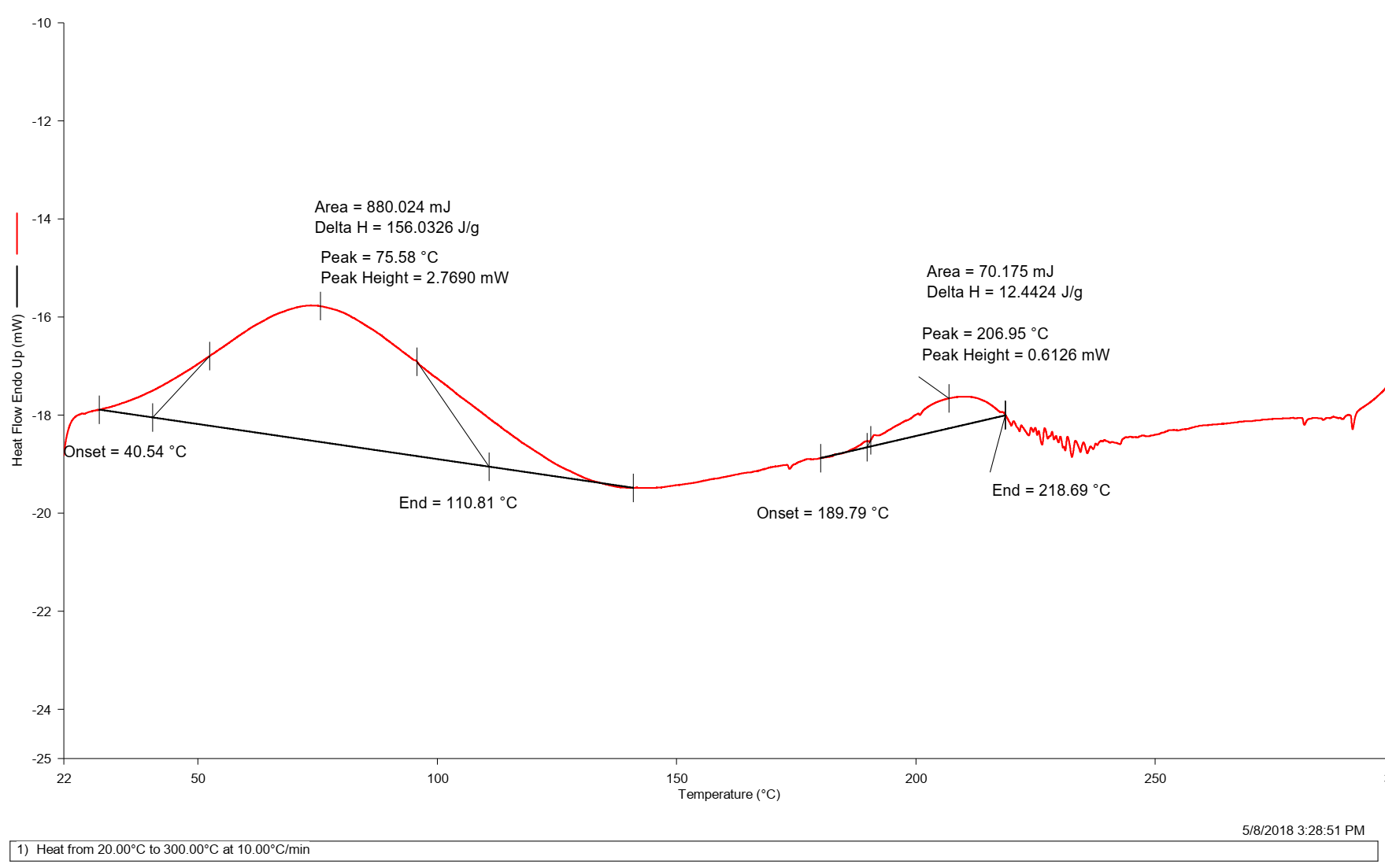


Figure 6. DSC thermogram of whey protein maltodextrin conjugated with ratio 1:3 (WPMD 1:3)


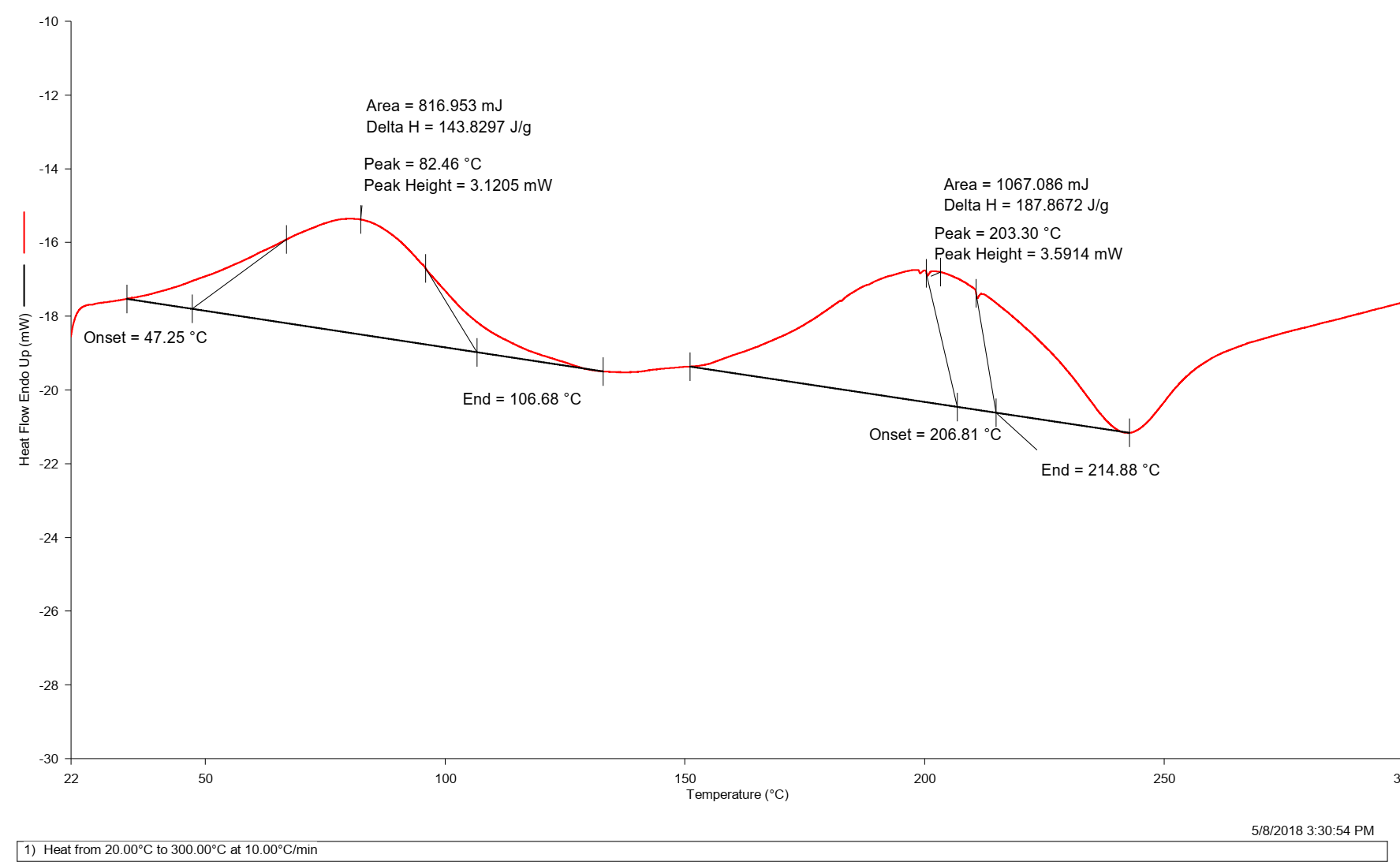


Figure 7. DSC thermogram of whey protein pectin conjugated with ratio 1:2 (WPP1:2)


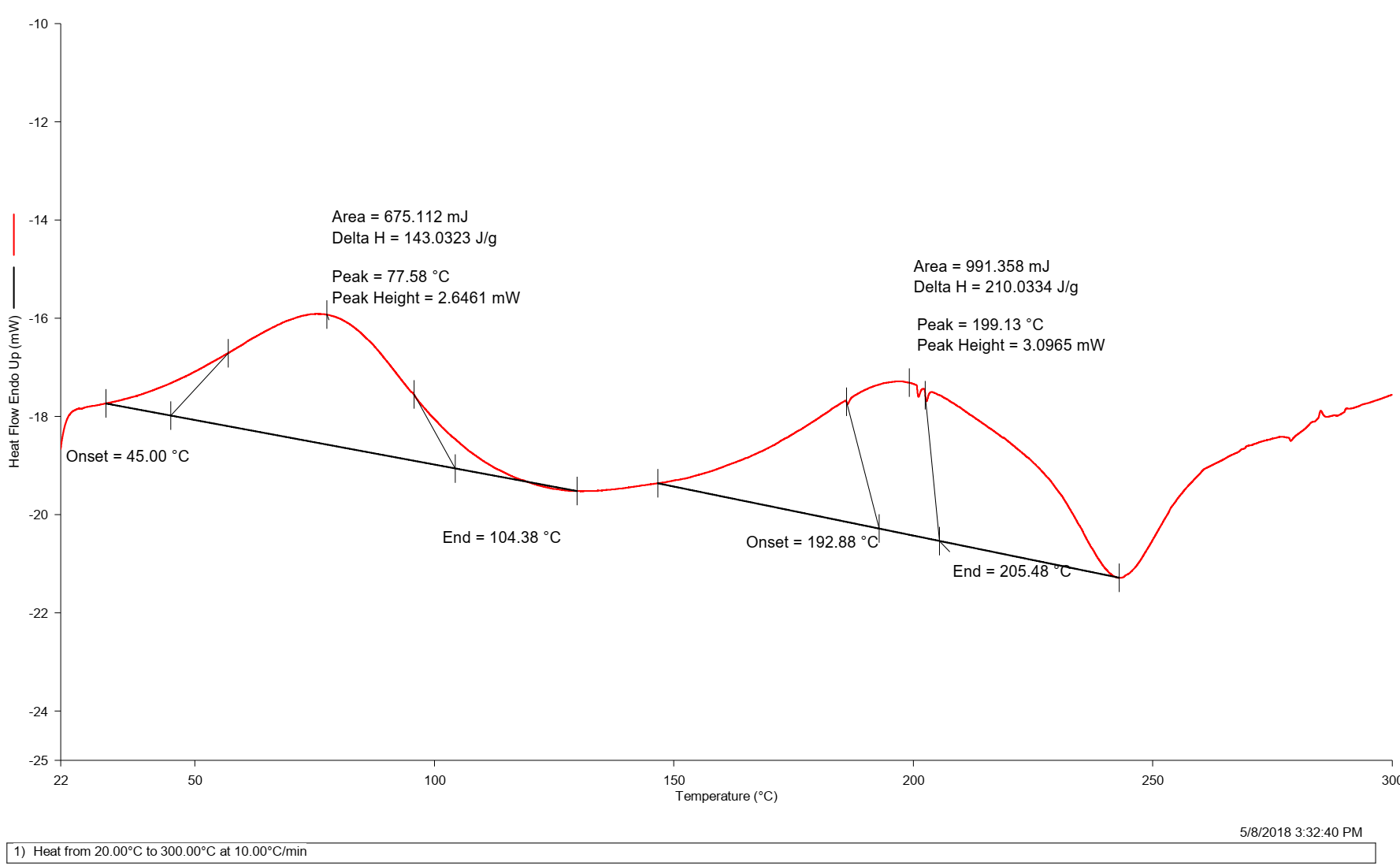


Figure 8. DSC thermogram of whey protein pectin conjugated with ratio 1:3 (WPP1:3)


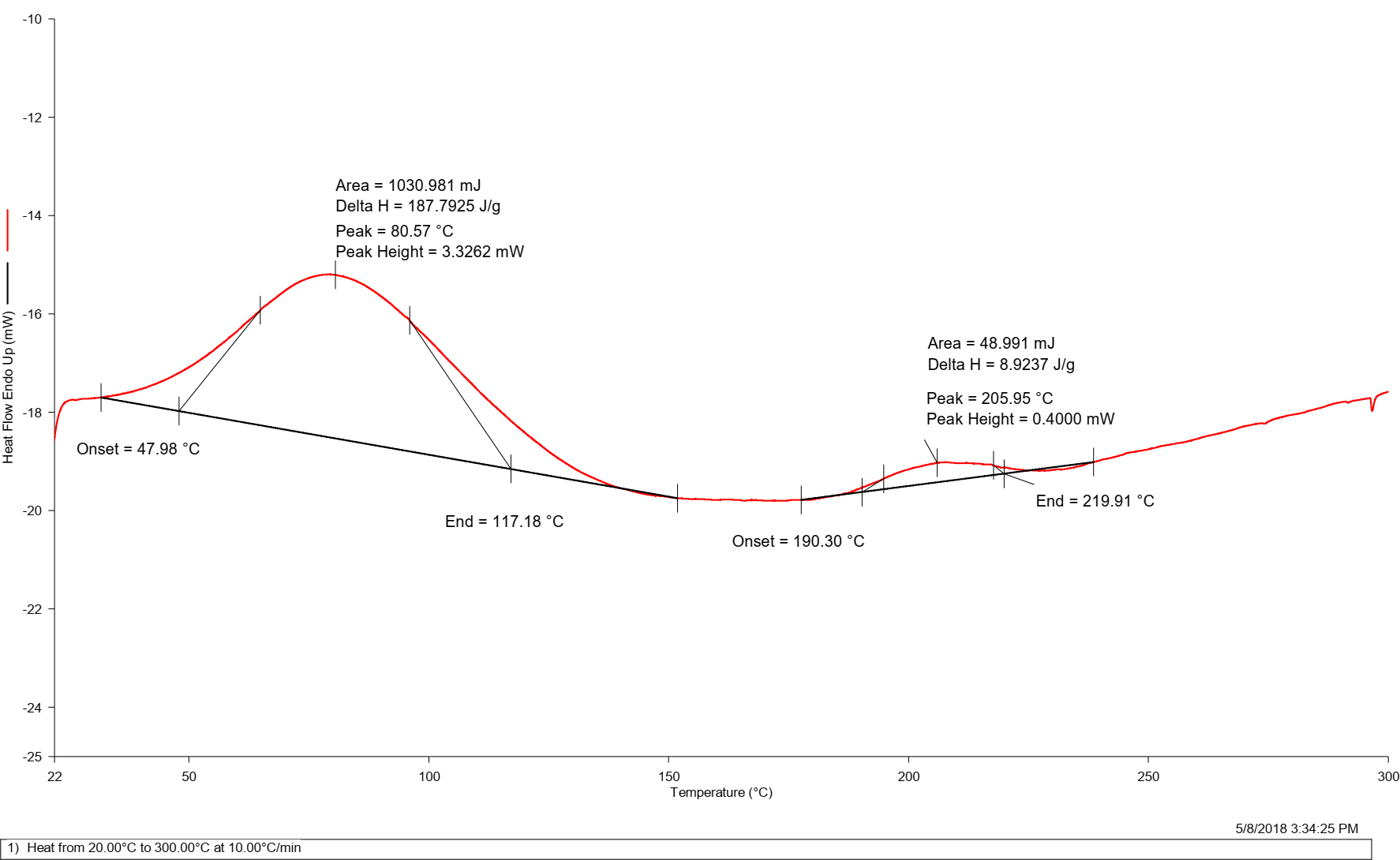


Figure 9. DSC thermogram of soy protein matodextrin conjugated with ratio 1:2 (SPMD1:2)


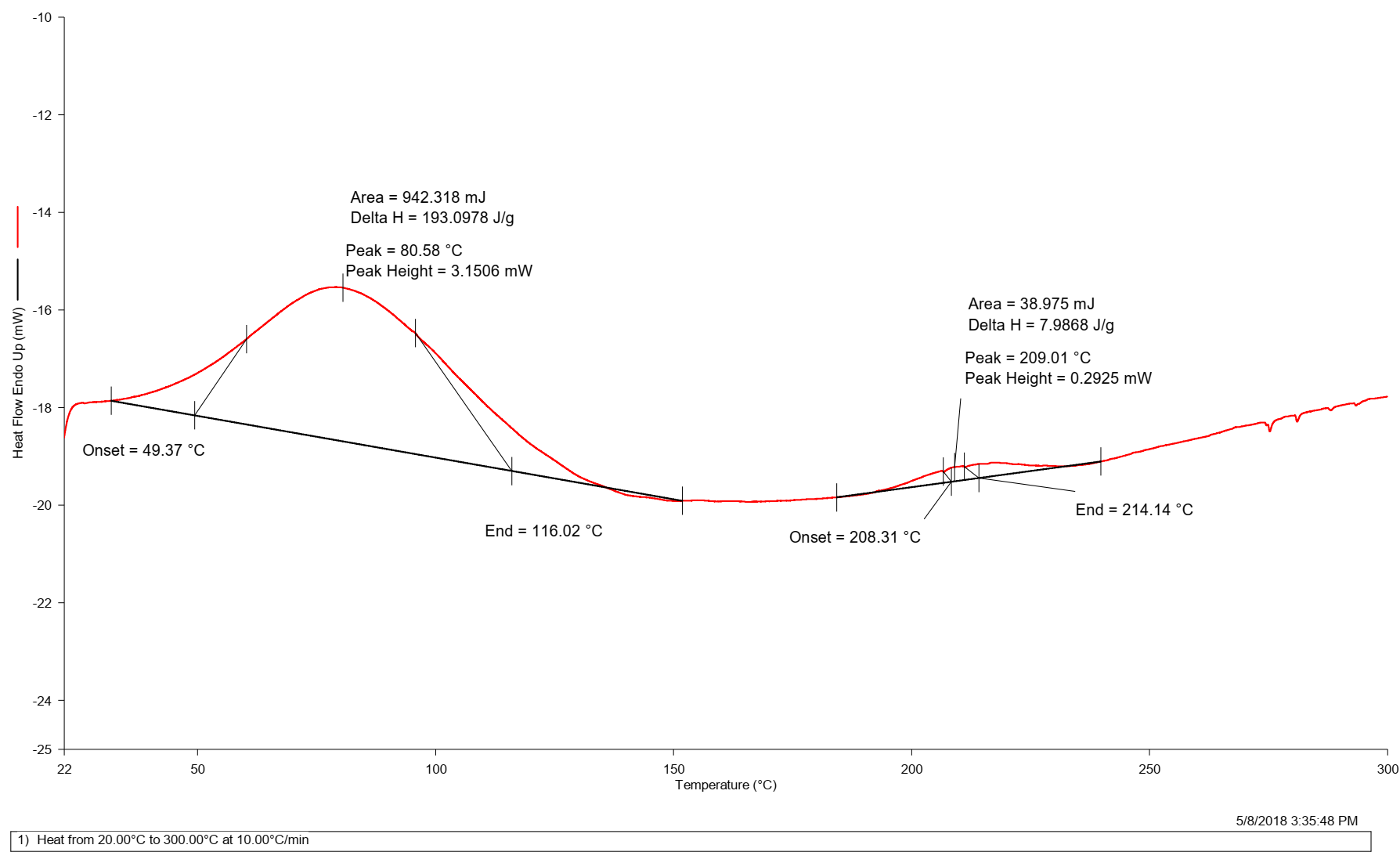


Figure 10. DSC thermogram of soy protein matodextrin conjugated with ratio 1:3 (SPMD1:3)


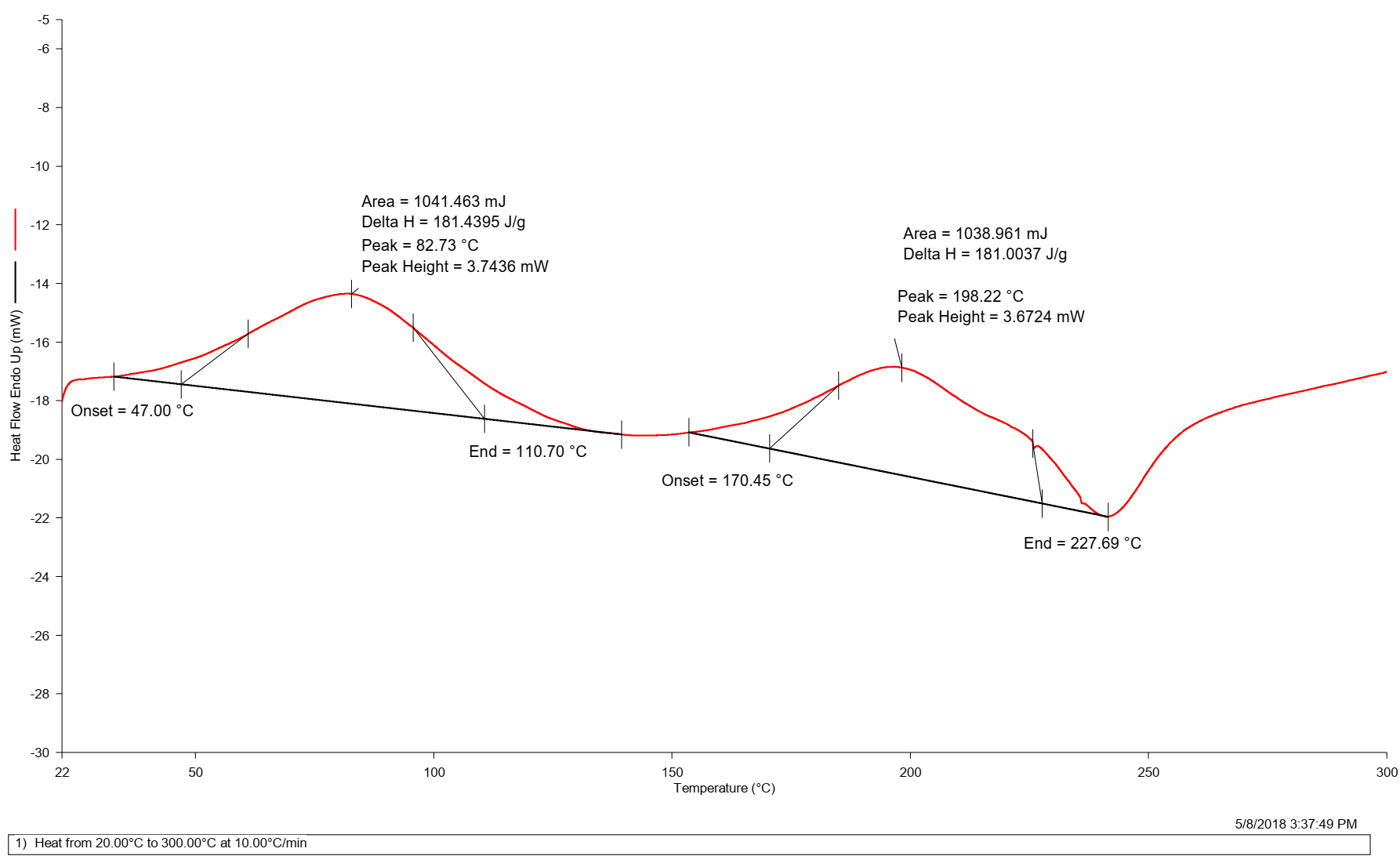


Figure 11. DSC thermogram of soy protein pectin conjugated with ratio 1:2 (SPP1:2)


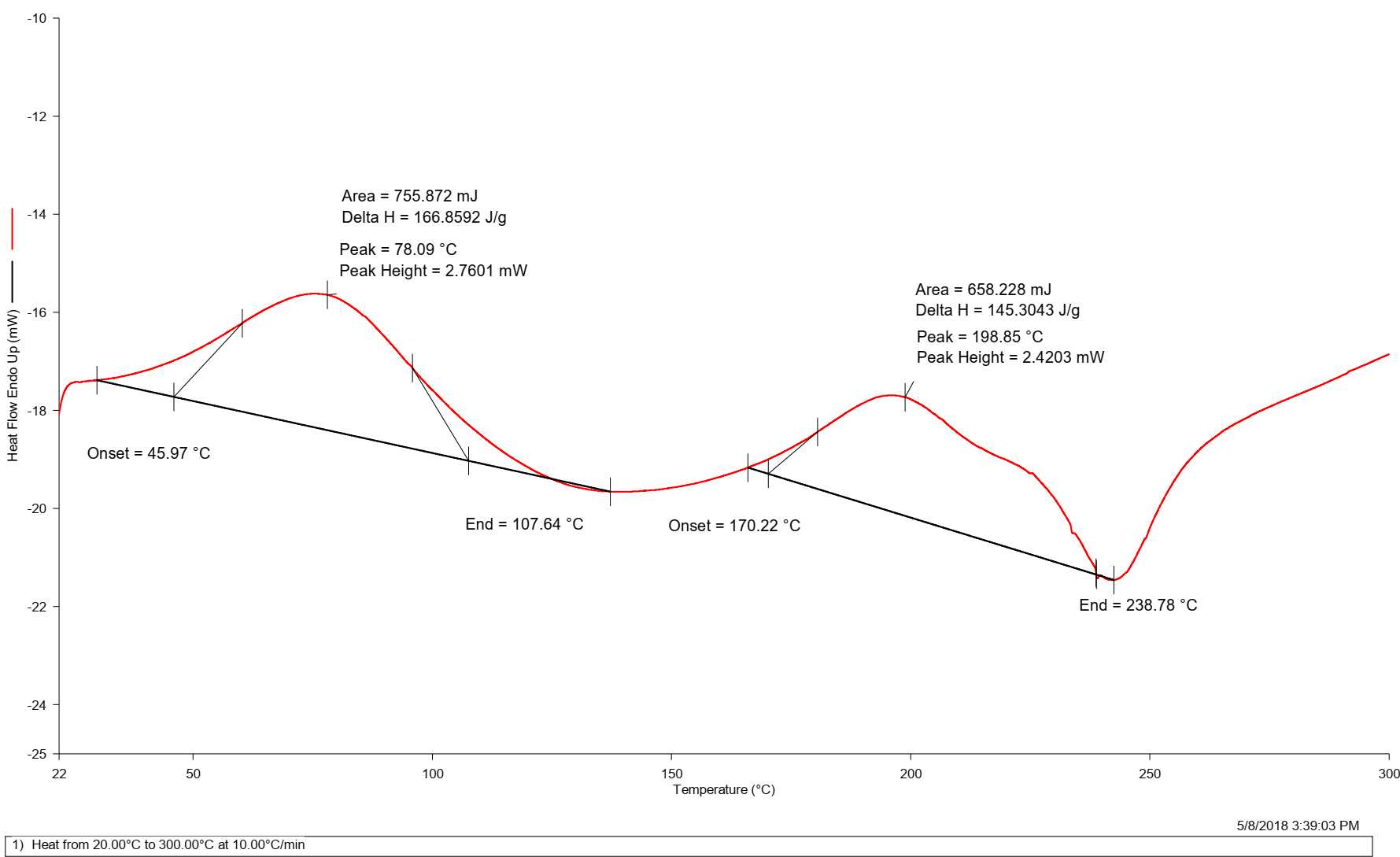


Figure 12. DSC thermogram of soy protein pectin conjugated with ratio 1:3 (SPP1:3)


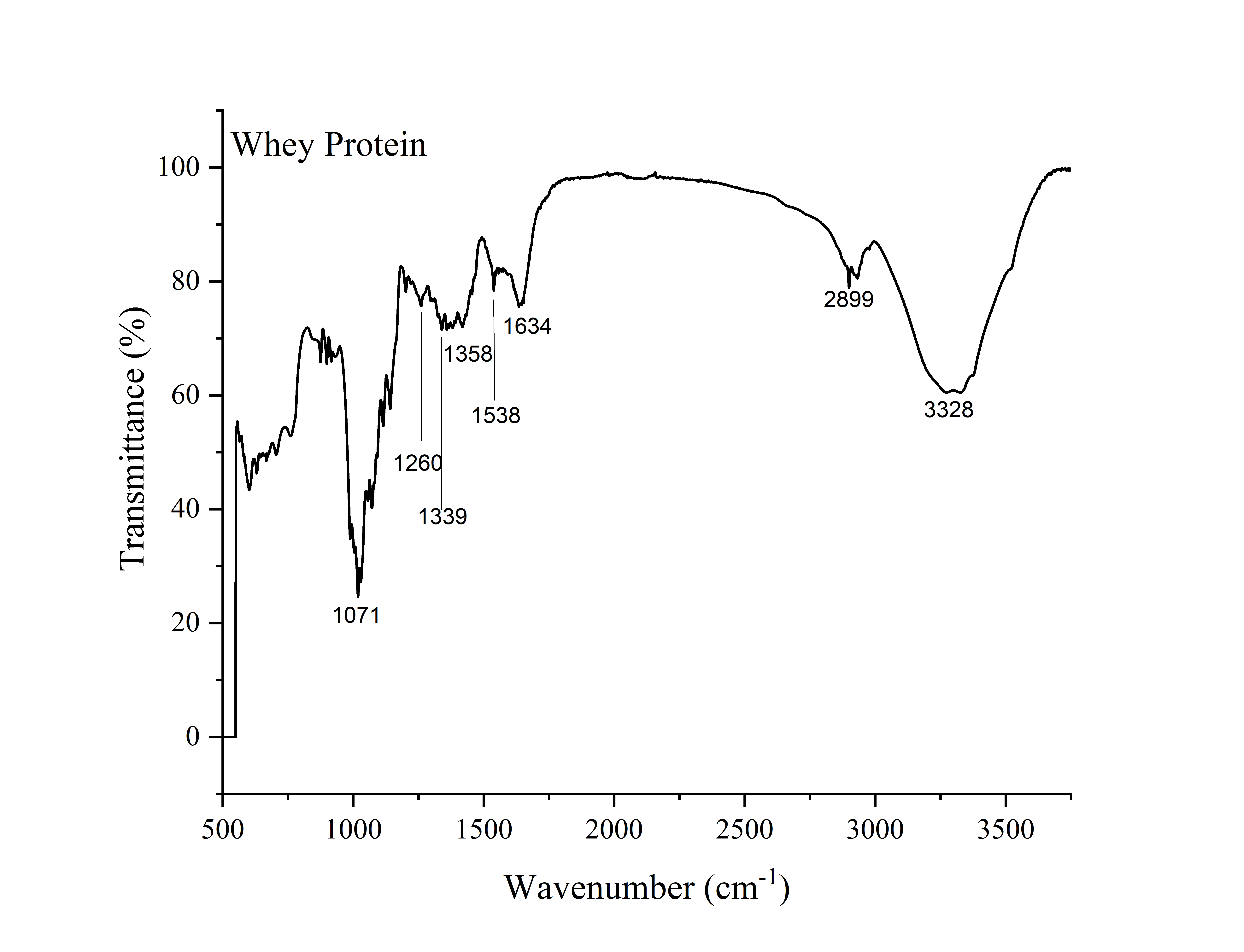


Figure 13. FTIR spectra of whey protein


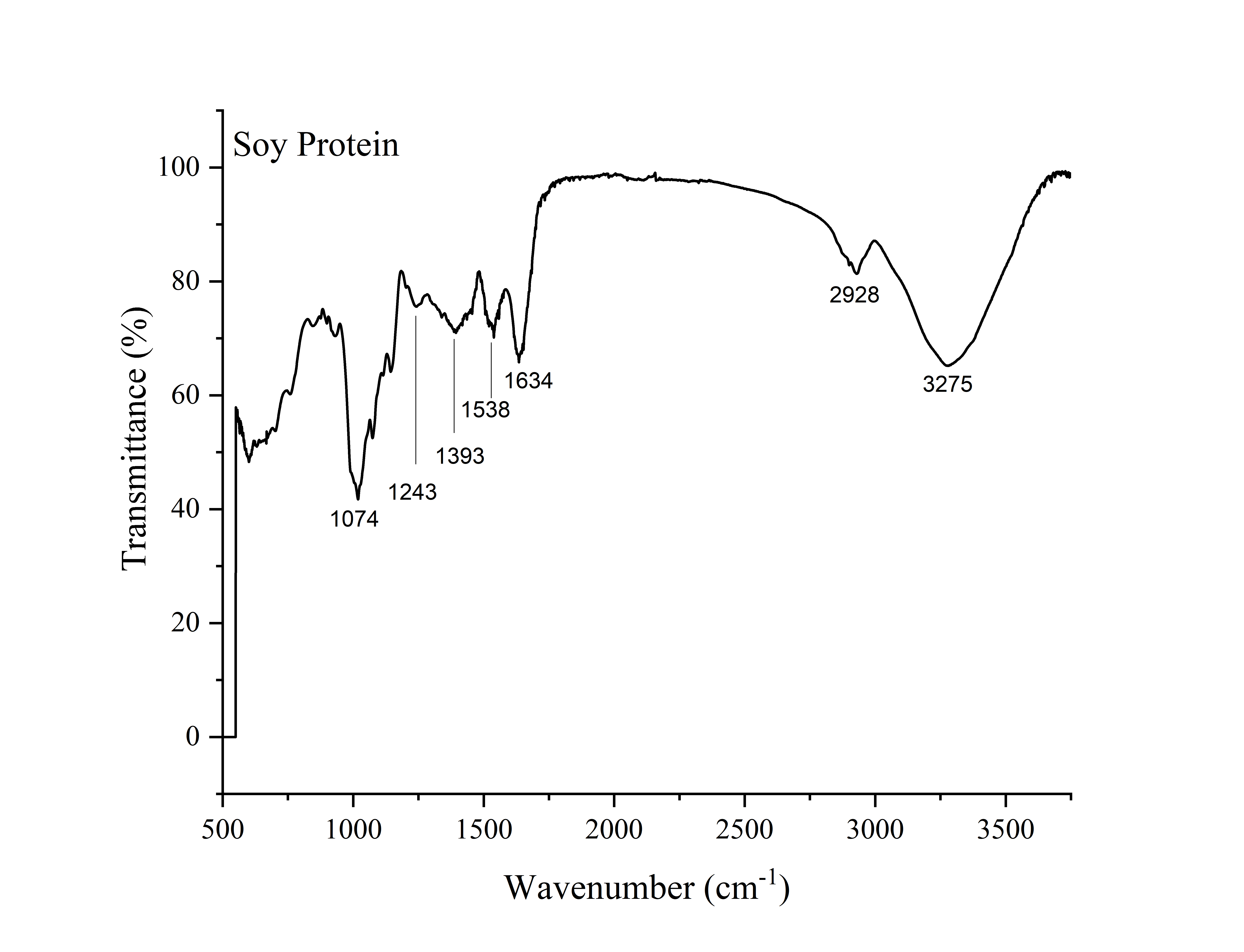


Figure 14. FTIR spectra of soy protein


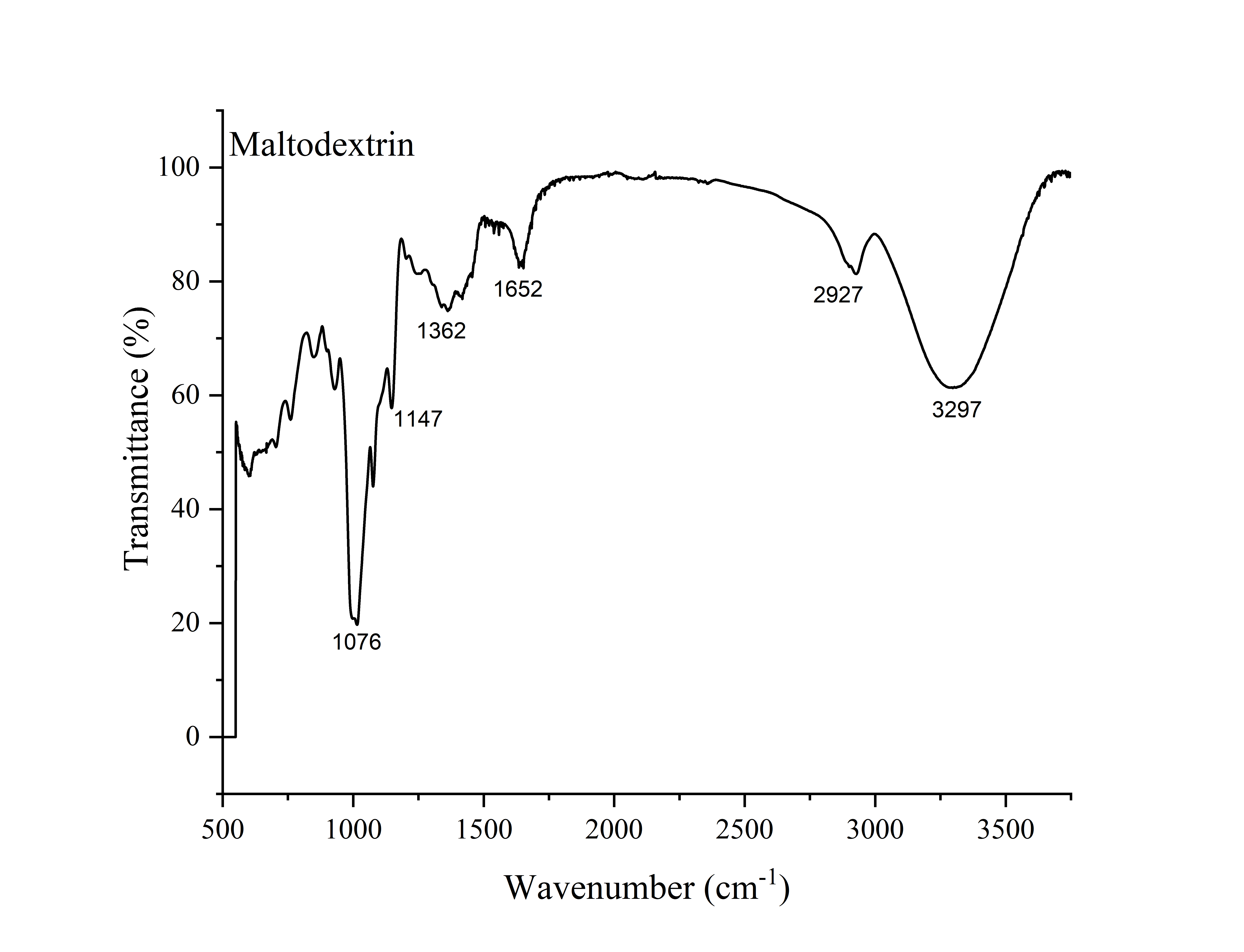


Figure 15. FTIR spectra of maltodextrin


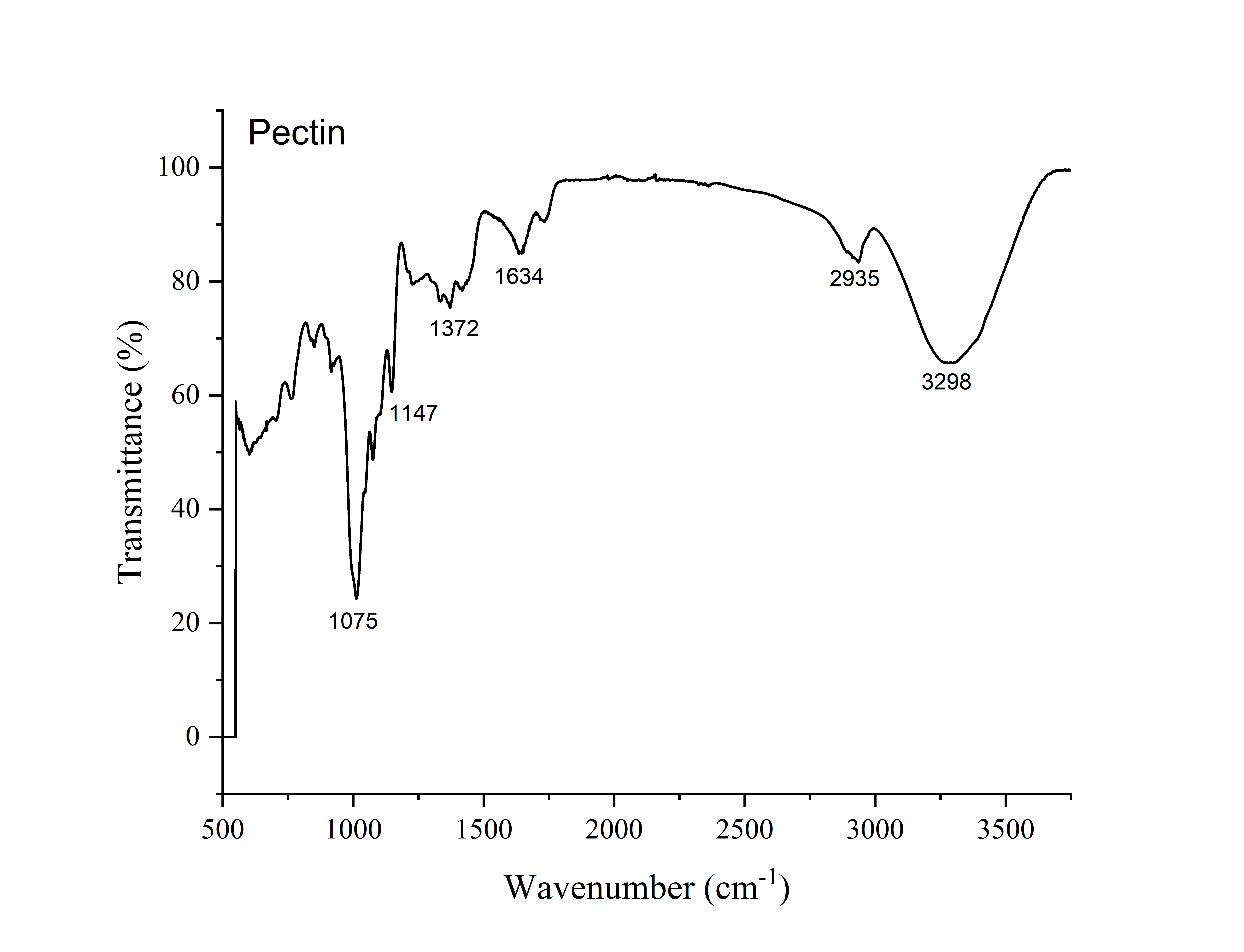


Figure 16. FTIR spectra of pectin
